# An advanced metabolomic approach on grape skins untangles cultivar preferences by *Drosophila suzukii* for oviposition

**DOI:** 10.3389/fpls.2024.1435943

**Published:** 2024-08-21

**Authors:** Rémy Marcellin-Gros, Sébastien Hévin, Clara Chevalley, Julien Boccard, Valérie Hofstetter, Katia Gindro, Jean-Luc Wolfender, Patrik Kehrli

**Affiliations:** ^1^ Institute of Pharmaceutical Sciences of Western Switzerland, University of Geneva, Geneva, Switzerland; ^2^ School of Pharmaceutical Sciences, University of Geneva, Geneva, Switzerland; ^3^ Research Division of Plant Protection, Agroscope, Nyon, Switzerland

**Keywords:** vinegar fly, mass spectrometry, metabolite profiling, molecular networks, AMOPLS, flavonoids, cuticle, integrated pest management. article types: original research

## Abstract

Insects’ host preferences are regulated by multiple factors whose interactions are only partly understood. Here we make use of an in-depth, untargeted metabolomic approach combining molecular networking (MN) and supervised Analysis of variance Multiblock Orthogonal Partial Least Squares (AMOPLS) to untangle egg-laying preferences of *Drosophila suzukii*, an invasive, highly polyphagous and destructive fruit pest originating from Southeast Asia. Based on behavioural experiments in the laboratory as well as field observation, we selected eight genetically related *Vitis vinifera* cultivars (e.g., Ancellotta, Galotta, Gamaret, Gamay, Gamay précoce, Garanoir, Mara and Reichensteiner) exhibiting significant differences in their susceptibility toward *D. suzukii*. The two most and the two least attractive red cultivars were chosen for further metabolomic analyses of their grape skins. The combination of MN and statistical AMOPLS findings with semi-quantitative detection information enabled us to identify flavonoids as interesting markers for differences in the attractiveness of the four studied grape cultivars towards *D. suzukii*. Overall, dihydroflavonols were accumulated in unattractive grape cultivars, while attractive grape cultivars were richer in flavonols. Crucially, both dihydroflavonols and flavonols were abundant metabolites in the semi-quantitative analysis of the extracted molecules from the grape skin. We discuss how these two flavonoid classes might influence the egg-laying behaviour of *D. suzukii* females and how they could serve as potential markers for *D. suzukii* infestations in grapes that can be potentially extended to other fruits. We believe that our novel, integrated analytical approach could also be applied to the study of other biological relationships characterised by multiple evolving parameters.

## 1 Introduction

Europe and North America are confronted since 2010 with the invasion of the spotted wing *Drosophila suzukii* Matsumura (Diptera: Drosophilidae), a highly polyphagous and destructive fruit pest originating from Southeast Asia (Lee et al., 2011a, 2011b; Walsh et al., 2011; Wang et al., 2016). Females’ large and serrated ovipositor enables them to lay their eggs in intact, ripening fruits (Atallah et al., 2014; Poyet et al., 2014). Endemic *Drosophila* species from Europe and America lack such a pronounced ovipositor and they are, therefore, mostly laying their eggs in decaying and rotten fruits. The oviposition of eggs in ripening fruits shortly before harvest makes *D. suzukii* a major pest in fruit production, leading to severe economic losses within the fruit industry (Burrack et al., 2013; Rota-Stabelli et al., 2013; Cini et al., 2014; Ioriatti et al., 2015; Keesey et al., 2015).

*Drosophila suzukii* has a very wide range of potential host fruits with more than 200 plant species from at least 40 different plant families identified for egg-laying (Mitsui et al., 2010; Cini et al., 2012; Baroffio et al., 2014; Poyet et al., 2014; Lee et al., 2015; Arnó et al., 2016; Kenis et al., 2016). Besides a large range of non-crop host plants (Kenis et al., 2016), *D. suzukii* also attacks cultivated crops of thin-skinned berries (e.g., raspberries, blueberries, blackberries, strawberries), stone fruits (e.g., cherries, peaches, apricots, plums) (Bellamy et al., 2013; Asplen et al., 2015) as well as grapes (Ioriatti et al., 2015). Overall, oviposition preferences by *D. suzukii* females vary with the range of available host fruits. In general, female flies prefer thin-skinned berries over thick-skinned fruits as highlighted in a multi-choice experiment by Cai et al., 2019 but they can also infest less suitable fruits when other favourable plant species are missing. Although it is widely accepted that these host preferences are regulated by multiple factors, such as olfactive, visual cues and textural aspects, their interactions are only partly understood.

Plant volatiles play an important role in the identification of suitable host plants (Bruce and Pickett, 2011). They are therefore important for insects to localise potential food sources or oviposition sites (Stensmyr et al., 2012). In addition, skin hardness affects the egg-laying capacity of *D. suzukii* females (Lee et al., 2016; Entling et al., 2019; Shrader et al., 2019). Typically, unripe and hard fruits are not attractive to *D. suzukii* for oviposition and fruits’ susceptibility increases as they ripen (Lee et al., 2011a). In grapes and fruits in general, *D. suzukii* oviposition steadily rises from veraison to harvest, coinciding with increasing sugar levels, decreasing acidity and softer berries (Ioriatti et al., 2015). Moreover, most fruits also become darker during the ripening process (Castellarin et al., 2016) through an accumulation of anthocyanins (Boss et al., 1996). Females of *D. suzukii* therefore commonly prefer red and dark fruits over brightly coloured ones (Rice et al., 2016). In the case of grapes, more eggs are generally laid in red than white cultivars (Linder et al., 2014).

Fruits are typically covered with a cuticle that might alter the physical and chemical properties of their surface. The thickness and chemical composition of the fruits’ cuticle differ across plant species and varieties (Weißinger et al., 2021). The cuticle contributes to the protection of fruits against multiple abiotic and biotic stresses such as drought, UV radiation, frost, fungal pathogens or phytophagous arthropods (Gindro and Pezet, 1999; Schnee et al., 2008; Arya et al., 2021). The cuticle formation influences attractiveness and vulnerability of fruits to *D. suzukii* infestation (Weißinger et al., 2021). Although multiple factors have been investigated to understand a fruits’ attractiveness and predict their vulnerability, none of them has so far been identified as the key parameter regulating *D. suzukii* infestation. To our knowledge, few studies have examined how the composition and metabolomic content of fruit skins influence the oviposition behaviour of *D. suzukii* females until today (Wang et al., 2022; Olazcuaga et al., 2023). Moreover, there are still missing reliable chemical markers that might indicate the susceptibility of a fruit to *D. suzukii* infestation. Metabolomic analysis might help to untangle these open questions since they aim to quantify all metabolites and identify potential markers in a cellular system in order to understand an organism’s physiological response to intrinsic and extrinsic factors (Johnson et al., 2016).

In this study we aim to investigate oviposition preferences of *D. suzukii* among different grape cultivars (*Vitis vinifera* L.) at different ripening stages with a particular emphasis on the influence of the chemical composition of the berry skin. Although grapes are considerably less attractive to *D. suzukii* for egg-laying than for example cherries, strawberries or blueberries (Lee et al., 2011a; Bellamy et al., 2013; Burrack et al., 2013; Aly, 2018; Olazcuaga et al., 2019), *V. vinifera* is the only species that shows a wide range of susceptibility levels between its different cultivars. Some cultivars are almost neglected for egg-laying (e.g., Cabernet Sauvignon, Merlot, Galotta, Gamaret) while others are considered as highly attractive (e.g., Schiava, Gamay précoce, Mara) (Ioriatti et al., 2015; Kehrli and Linder, 2018). With this in mind, we selected eight genetically related grape cultivars and examined their attractiveness and susceptibility toward *D. suzukii* in laboratory assays supported by field observation. The two most and two least attractive cultivars were subsequently chosen for a comprehensive, untargeted metabolomic analysis of their grape skin to determine whether the differences in attractiveness among these cultivars could be attributed to variations in the chemical composition of the skin. For this purpose, we extracted the secondary metabolites of skins and determined their mass spectra. Aiming to identify markers that might be related to the observed differences in grape cultivars’ attractiveness for egg-laying of *D. suzukii*, we used a multivariate statistical approach dedicated to the analysis of multifactorial experiments, i.e., Analysis of variance Multiblock Orthogonal Partial Least Squares (AMOPLS).

## 2 Materials and methods

### 2.1 Natural oviposition preferences in the field

Based on previous surveys on the susceptibility of grape cultivars (Kehrli and Linder, 2018), grape bunches from eight cultivars genetically related to Gamay noir (=Gamay), Ancellotta and Reichensteiner (**Table 1**) were collected during their final ripening stages from veraison (BBCH 85 = softening of berries) to complete maturity (BBCH 89 = berries ripe for harvest) (Lorenz et al., 1995) in the grapevine cultivar collection of Agroscope in Pully (Switzerland) (**Supplementary Figure S1**). Together with Gamay noir, Ancellotta and Reichensteiner, five other grape cultivars, i.e., Galotta, Gamaret, Gamay précoce, Garanoir and Mara were tested and the close genetic relationship among each other emerges from their crossing partners stated in **Table 1**. All eight cultivars were sampled at different stages of grape maturation on four dates between August 26^th^ and September 24^th^, 2019. Two to three bunches of each grape cultivar were collected, placed in an airtight bag and transported in a cool box to the laboratory. Thirty berries were then randomly selected from each grape cultivar. The number of berries infested with *D. suzukii* eggs was counted under a stereomicroscope. The percentage of infested berries per cultivar was calculated for each of the four dates. However, as *D. suzukii* infestation was absent for the first three dates (e.g., August 26^th^, September 2^nd^ and 9^th^) so that only descriptive statistical analyses were performed for the last sampling on September 24^th^, 2019.

**Table 1:** Names of the grape cultivars used, with the breeding partners of the original cross-breeding (indicated by *), their country of origin as well as the year of crossing based on Maul et al. (2023): Vitis International Variety Catalogue - **www.vivc.de** - (08/2023).

Grape cultivar	Grape skin color	Crossing partners	Origin	Year of crossing
**Gamay noir**	Red	Heunisch Weiss ^W^ * Pinot noir ^R^	France	
**Reichensteiner**	White	Müller Thurgau Weiss ^W^ * Madeleine angevive × Calabre ^R^	Germany	1978^1^
**Ancellotta**	Red	Unknown	Italy	~1700
**Gamay précoce**	Red	Heunisch Weiss ^W^ * Pinot noir ^R^	France	
**Mara**	Red	Gamay noir ^R^ * Reichensteiner ^W^	Switzerland	1970
**Gamaret**	Red	Gamay noir ^R^ * Reichensteiner ^W^	Switzerland	1970
**Garanoir**	Red	Gamay noir ^R^ * Reichensteiner ^W^	Switzerland	1970
**Galotta**	Red	Ancellotta ^R^ * Gamay noir ^R^	Switzerland	1981

^R^ Indicates grape cultivars of red berry skin.

^W^ Indicates grape cultivars of white berry skin.

^1^ Indicates the year in which the grape cultivar was protected.

### 2.2 Attractiveness of grape cultivars towards *D. suzukii* in a multiple-choice laboratory experiment

All *D. suzukii* adults used in the multiple-choice laboratory experiment were reared on a homemade growing medium consisting of mashed banana peel, agar, brewer’s yeast, wheat flour, sugar, methylparaben, alcohol and water in contact with circa 50 to 200 conspecifics in a common rearing. Flies used in this multiple-choice experiment were between three and five days old and it was assumed that a large majority of females had mated before the start of the experiment or was likely to do so during the experiment.

A grape bunch was collected from each grape cultivar from which intact and uninfested berries were selected through inspection under stereomicroscope. Thereafter each berry was weighed and placed individually on the lid of a petri dish of 3.5 cm diameter. One lid with a single berry of each of the eight cultivars was exposed in a rectangular box (25 × 15 × 8 cm) to 15 naïve females and 10 naïve males of *D. suzukii*. In addition, a small feeder consisting of a plastic box of 1 cm^3^ filled with sugar water and accessible to the flies through a dental cotton roll was placed in each of the rectangular boxes to ensure that flies remained alive over the duration of the experiment. After introducing the flies and the feeder, boxes were closed with a mesh lid to ensure air ventilation and prevent condensation. Boxes were then stored in a climate chamber at 22°C, 75% RH and 16/8 day/night for 48 hours. The position of the berries of the eight cultivars within the box and the place of a box within the climate chamber were arbitrarily varied. After 48 hours, *D. suzukii* flies were removed and the number of eggs laid on each berry of the eight cultivars was counted using a stereomicroscope. At each of the four sampling dates, six plexiglass boxes were set up in this multiple-choice laboratory experiment resulting in a total of six repetitions per date.

To standardize oviposition the number of *D. suzukii* eggs laid on a berry was divided by the berry’s initial weight resulting in the ‘number of eggs per gram of fruit’. For each cultivar in a plexiglass box, the ‘number of eggs per gram of fruit’ was used as the dependent variable in the following statistical analyses, whereas ‘box’, ‘cultivar’ and ‘date’ were treated as nominal independent factors. Data were analysed using Multivariate analysis of variance with R 4.2.3 using the two packages multcompView and ggpubr. Potential statistical differences among the eight cultivars were calculated using Bonferroni pairwise comparisons. The ‘number of eggs per gram of fruit’ was log-transformed (log[x+1]) to fit normal distribution, and the fulfilment of the model assumptions was checked by visually inspecting the distribution of the residuals.

### 2.3 Preparation of grape skin for metabolomic analyses

For each date and each cultivar, berries without eggs and wounds were selected and peeled with a scalpel to obtain their skins. The skins were finely ground with a mortar in liquid nitrogen and stored in vials at -80°C. Samples were then freeze-dried and about 30 mg were collected and solubilized into 2.2 mL of hexane in order to remove fats. To do this, samples were vortexed, sonicated for 15 min, re-vortexed and finally centrifuged for 10 min at 5’000 rpm. The supernatant enriched in fats was discarded and the pellet was dried and resolubilized in 2.2 mL of dichloromethane. Once more, the samples were vortexed, then sonicated for 15 min, centrifuged for 10 min at 5’000 rpm and 2.0 mL were collected in a tared vial and subsequently dried. These 2.0 mL of grape skin formed the dichloromethane extracts.

In a second step, the previously insolubilized material was resolubilized in 2.2 mL of methanol, vortexed, sonicated for 15 min at 40°C and centrifuged for 10 min at 5’000 rpm. 2.0 mL were collected in tared vial, dried and then freeze-dried for 4.5 hours. These 2.0 mL of grape skin constituted the methanol extracts.

### 2.4 UHPLC-PDA-CAD-HRMS/MS analyses

Methanolic and dichloromethane grape skin extracts were analysed on a Waters Acquity UPLC I-Class system (Waters^®^, Milford, USA) coupled to a Thermo Scientific Orbitrap Exploris 120 mass spectrometer (Thermo Scientific^®^, Bremen, Germany), using a Thermo Scientific OptaMax NG ion source with a heated electrospray ionization (HESI-II) probe. The liquid chromatographic conditions were set as follows: the column was a Waters BEH (Ethylene Bridget Hybrid) C18 50 × 2.1 mm, 1.7 μm; the mobile phase was (A) water and (B) acetonitrile both with 0.1% formic acid and the gradient was as follows: linear from 5 to 100% of B over 7 min and isocratic at 100% B for 2 min followed by 1 min equilibration at 5% of B; the flow rate was set to 600 μL/min for an injection volume of 6 μL; a Waters Acquity UPLC photodiode array (PDA) detector was used to acquire the PDA spectra collected on a wavelength range from 210 nm to 400 nm. Thereafter, two-thirds of the flow was diverted to a Thermo Scientific charged aerosol detector (CAD) Corona ultra RS for quantification and one third to the mass spectrometer. The optimized HESI-II parameters were as follows: source voltage was set from 3.1 kV to 3.7 kV from 1 min to 6 min and maintained at 3.7 kV until 10 min in positive mode and 2.5 kV in negative mode; sheath gas flow rate (N2), 35 units; auxiliary gas flow rate, 10 units (pos) and 7 units (neg); sweep gas flow rate, 1.0; ion transfer tube temperature and vaporizer temperature were set to 320°C and 320°C respectively in positive mode and 310°C and 290°C respectively in negative mode. The mass analyser was calibrated using the Thermo Scientific EASY-IC ion source internal reference mass (fluoranthene). The mass spectrometer method was set to FullMS data-dependent MS2 (ddMS2) for a scan range between 100 to 1’500 *m/z*. FullMS were acquired at a resolution of 30’000 for an expected peak width of 16 s and the normalized AGC target was set to 350% and injection time was set to 120 ms. In ddMS2, the resolution was 15’000, the normalised AGC target 50%, the isolation window 1.5 *m/z*, and the stepped normalized collision energy 15/30/45. Injection time was set to 125 ms and the parent ions were placed on the dynamic exclusion list for 2 s. The RF lens was set on 60%. Again, the Thermo Scientific EASY-IC ion source internal reference was used for mass calibration.

### 2.5 LC-MS/MS data pre-processing

Thermo Scientific mass spectrometer raw files were first converted to mzML format with ThermoRawFileParser1.4.1 (Hulstaert et al., 2020) with the excludeExceptionData option to remove the fluoranthene signal (202.0778 *m/z*). MS data were processed using MZmine 3.4.27 software (Schmid et al., 2023). The main parameters were an intensity detection threshold for MS1 masses of 1.0^5^ and 0 for MS2; extracted ion chromatograms were reconstructed using the ADAP chromatogram builder module (Du et al., 2020) with a minimum scan group size of 5, a minimum intensity for consecutive scans of 3.0^5^, a minimum absolute height of 1.0^6^ and a scan-to-scan *m/z* tolerance of 3 ppm. Extracted ion chromatograms were smoothed using the Savitzky-Golay algorithm over 5 time points. The parameters used for the ADAP feature resolver were a minimum feature height of 1.0^5^, a S/N threshold of 10, in S/N estimator the intensity window SN option was checked with a coefficient/area threshold of 100 and a peak duration range of 0 to 0.5 min and a RT wavelet range of 0 to 0.05 min. The MS/MS scan pairing was completed, with a minimum relative feature height of 25%, a minimum required signal of 1, a MS1 to MS2 precursor tolerance of 3 ppm and a retention time filter using a tolerance of 0.2 min. ^13^C isotope filter module was used to remove isotopologues, *m/z* tolerance was set to 3 ppm and RT tolerance to 0.2 min. Each sample features list was aligned using the join aligner module with a *m/z* tolerance of 3 ppm and a RT tolerance of 0.1 min. Weights associated to *m/z* and RT parameters were set to 80 and 20, respectively. The gap filling module was applied to the aligned feature list. Finally, only features having associated MS2 spectra were filtered and kept for further analyses. The aligned feature quantification table (matrix of feature abundancies per samples) and the MS1/MS2 mass spectra data file (.mgf) were exported and used for further statistical analyses, notably the feature-based molecular network (MN) calculation and the compounds annotation.

### 2.6 Statistical analysis for metabolomics

Data from the aligned feature quantification table were normalised, transformed and scaled prior to statistical analyses through the MetaboAnalyst 5.0 platform (Lu et al., 2023) in order to remove sample preparation effects. Normalisation was performed at the sample level by the Probabilistic Quotient Normalization (PQN) method taking QC samples as reference (Dieterle et al., 2006). Unit variance was applied to give an equal chance to each variable to contribute to the multivariate models, independently of its intensity range. Considering the experimental design, a 2-way ANOVA decomposition was performed for each solvent (MeOH and DCM) and each ionisation mode (ESI+ and ESI-) to estimate the contribution of ‘sampling date’ and ‘cultivar’ compared to the total variability. The ANOVA submatrices were then processed through supervised multiblock algorithm based on the Orthogonal Partial Least Squares (OPLS) framework as developed by Boccard and Rudaz (2016) under MATLAB environment. Separate sample ‘scores’ and feature ‘loadings’ were calculated for each model component to assess the relationships between the measured signals and effects. More specifically ‘scores’ of samples and feature ‘loadings’ of the two first predictive components related to ‘cultivar’ main effect were analysed to highlight potential chemical markers linked to attractiveness. Bar charts were plotted in the R 4.2.3 environment using the ggplot2 3.4.2 package.

### 2.7 Feature-based molecular network

A feature-based molecular network (FBMN) (Nothias et al., 2020) was generated with the corresponding workflow on the GNPS platform (Wang et al., 2016). The GNPS parameters were set as follows: a precursor ion mass tolerance (PIMT) of 0.02 Da, a fragment ion mass tolerance (FIMT) of 0.02 Da and a minimum of 6 fragment ions in common and a cosine score threshold of 0.7. The FBMN graphs were generated with Cytoscape 3.10 (Shannon et al., 2003).

### 2.8 Metabolite annotations

Features were first tentatively annotated in an automated way with the SIRIUS 5.8.3 pipeline (Dührkop et al., 2019) starting from raw formula calculation, then structure elucidation by analysing MS2 spectra *via* the CSI: FingerID algorithm (Dührkop et al., 2015) and the reranking of candidate structures with the COSMIC workflow (Hoffmann et al., 2022). In addition, candidate structure results were refined by inspecting UV spectra to narrow metabolite chemical classification. MS2 spectra were also annotated by comparing them to experimental spectra from GNPS platform. Finally, the chemical class of candidates was assigned through NPClassifier implemented in the SIRIUS pipeline (Kim et al., 2021).

## 3 Results

### 3.1 Natural oviposition preferences in the field

**Figure 1:**
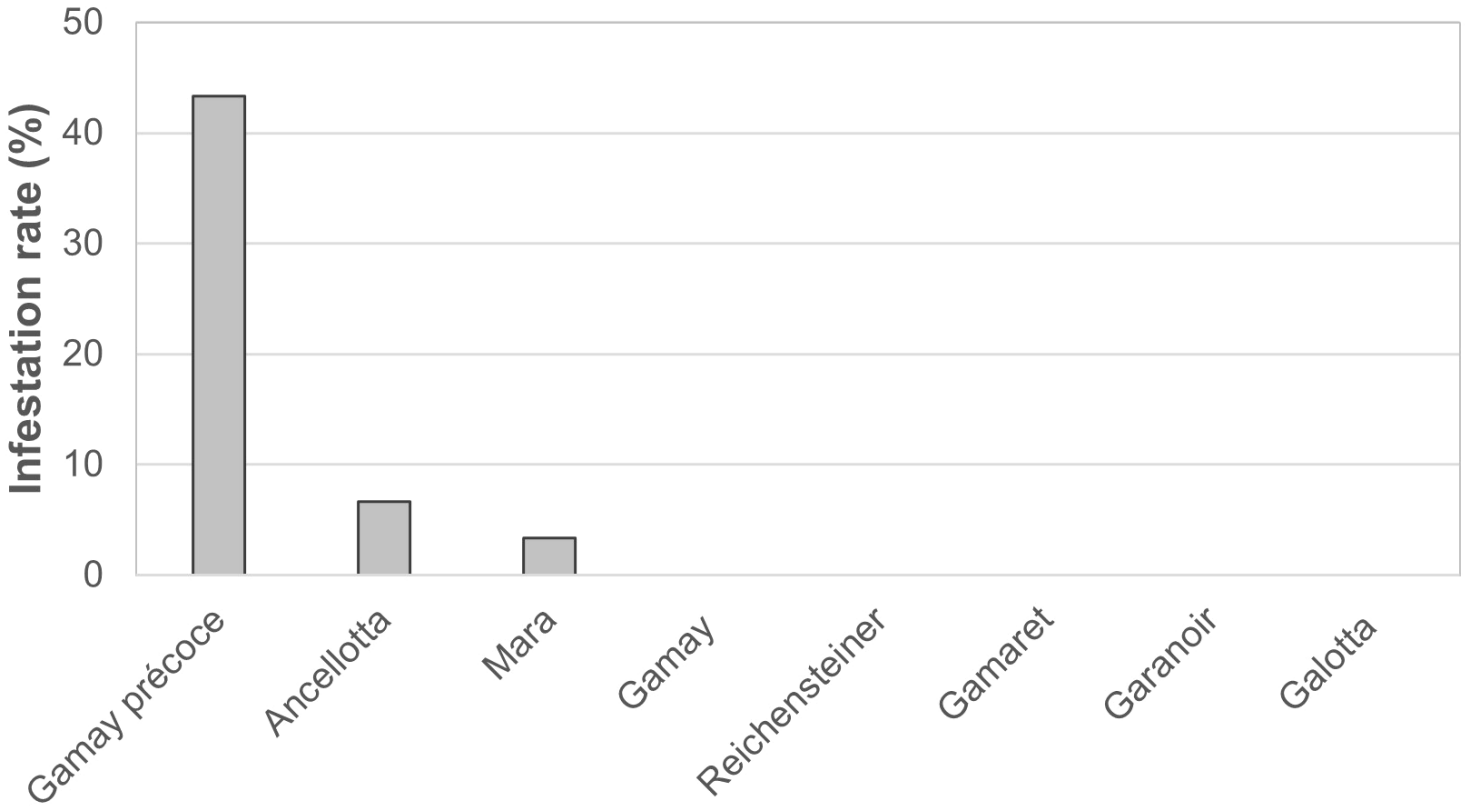
Percentage of berries infested with *D. suzukii* eggs for the eight cultivars in the experimental vineyard of Agroscope in Pully on September 24^th^, 2019.

With the aim of evaluating differences in the natural attractiveness of the eight grape cultivars for *D. suzukii* egg-laying, the progression of infestation was monitored in the Agroscope vineyard of Pully. No eggs of *D*. *suzukii* were observed in berries of the three first sampling dates. On September 24, 2019, the infestation rate was notably higher in Gamay précoce than the other seven cultivars, with 13 out of 30 berries being infested, resulting in an infestation rate of 43.3% (**Figure 1**). *Drosophila suzukii* also laid eggs in grape bunches of Ancellotta and Mara with 2 and 1 out of the 30 berries being infested, respectively. No eggs were observed in the berries of the other five cultivars (**Figure 1**).

### 3.2 Attractiveness of grape cultivars towards *D. suzukii* in a multiple-choice laboratory experiment

**Figure 2:**
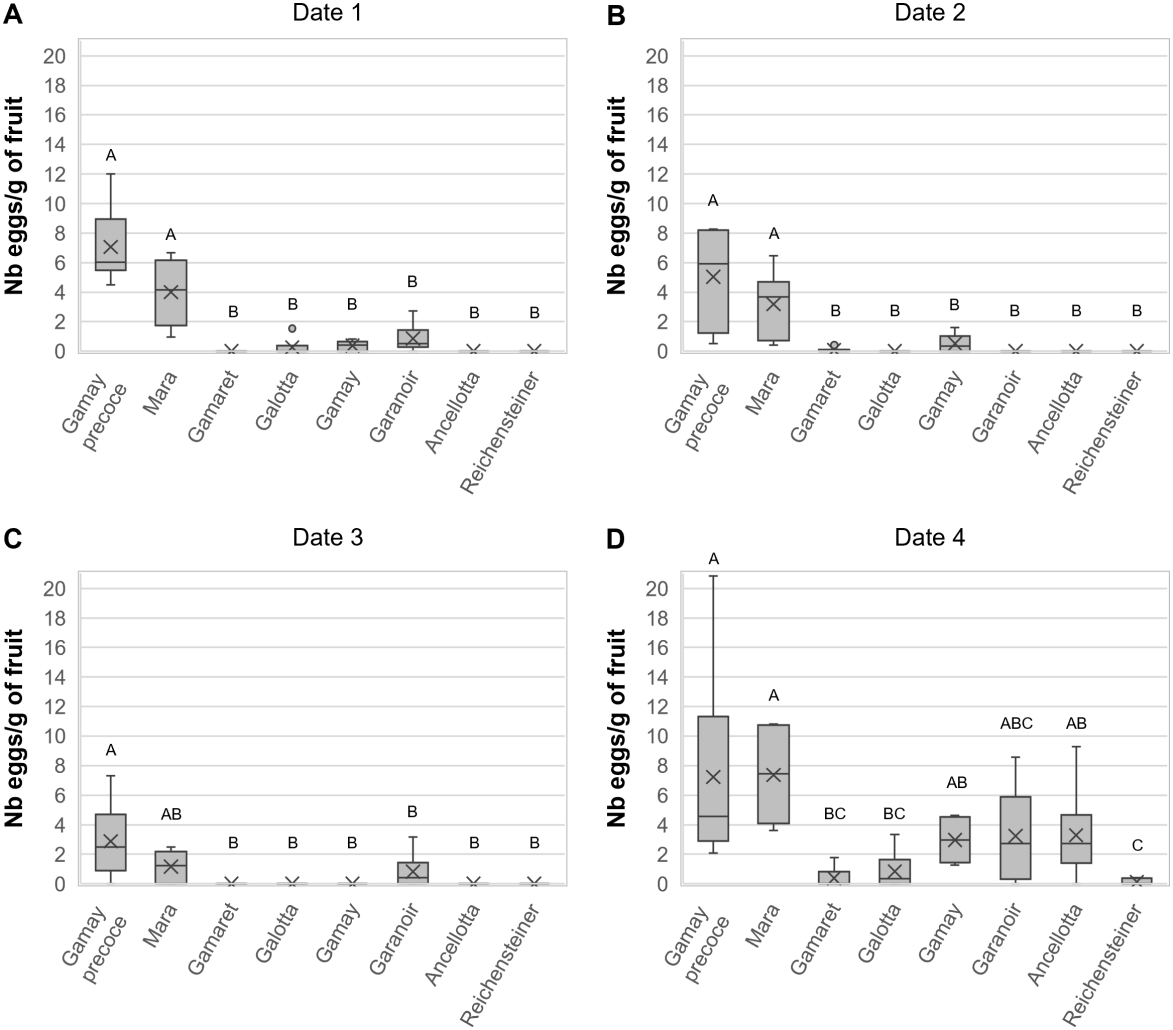
Boxplot diagrams on the number of *D suzukii* eggs per gram of fruits in the laboratory multiple-choice experiment on **(A)** August 26^th^, **(B)** September 2^nd^, **(C)** September 9^th^ and **(D)** September 24^th^, 2019. Boxplots indicated with different letters within a graph were significantly different in Bonferroni pairwise comparisons (p < 0.05).

After having tested the attractiveness of the eight cultivars in the vineyard, field observations were confirmed in the laboratory using a multiple-choice experiment presenting the eight cultivars simultaneously in a single box under standardised conditions. The overall analysis of the eight grape cultivars for the four dates in the year 2019 showed that there was no statistical difference between the different experimental ‘boxes’ with respect to the ‘number of eggs per gram of fruit’ laid (**Table 2**). However, the ‘cultivar’ of grapes as well as the ‘date’ of exposure had a significant effect on the oviposition behaviour of *D. suzukii* females (**Table 2**). Moreover, the statistical interaction between the eight ‘cultivars’ and the four ‘dates’ was highly significant (**Table 2**) indicating that the attractiveness of grape cultivars towards *D. suzukii* changed over time. It was thus decided to analyse the four dates separately. At all four dates, grape ‘cultivar’ had a highly significant effect on the ‘number of eggs per gram of fruit’ laid, whereas the factor ‘box’ remained non-significant (**Table 2**). At the first and second sampling dates on August 26^th^ and September 2^nd^, respectively, females of *D. suzukii* laid significantly more eggs in Gamay précoce and Mara compared to the other six cultivars (**Figures 2A, B**). On the third sampling date, Gamay précoce and Mara remained the most attractive grape cultivars, but only the first differed significantly from the other six (**Figure 2C**). On the fourth date on September 24^th^, three groups of attractiveness could be identified (**Figure 2D**). The most attractive group remained Gamay précoce and Mara, and these two cultivars were significantly preferred over Galotta, Gamaret, and Reichensteiner, on which nearly no eggs were laid. An intermediate level of attractiveness was observed for Gamay, Garanoir and Ancellotta. These three grape cultivars did not show significant differences from Gamay précoce, Mara, Galotta, and Gamaret. However, Gamay and Ancellotta were significantly more attractive than Reichensteiner (**Figure 2D**). September 24^th^ was the date where overall the highest ‘number of eggs per gram of fruit’ were laid, whereas oviposition was lowest on September 9^th^, the third sampling date (**Figure 2C**).

**Table 2:** Effects of ‘box’, ‘cultivar’ and ‘date’ on the ‘number of eggs per gram of fruit’ analysed with a 3-way ANOVA.

	Df	Sum Sq	Mean Sq	F value	*p* (>F)
All 4 dates:					
**Box**	20	3.80	0.19	1.06	0.40
**Cultivar**	7	63.61	9.09	50.54	< 0.001
**Date**	3	16.48	5.49	30.56	< 0.001
**Cultivar × Date**	21	10.25	0.49	2.72	< 0.001
**Residuals**	140	25.17	0.18		
T1 (26/08):					
**Box**	5	0.86	0.17	1.99	0.10
**Cultivar**	7	25.63	3.66	42.24	< 0.001
**Residuals**	35	3.03	0.09		
T2 (02/09):					
**Box**	5	0.82	0.16	1.11	0.37
**Cultivar**	7	17.85	2.55	17.35	< 0.001
**Residuals**	35	5.14	0.15		
T3 (09/09):					
**Box**	5	1.09	0.22	1.63	0.18
**Cultivar**	7	8.00	1.14	8.55	< 0.001
**Residuals**	35	4.68	0.13		
T4 (24/09):					
**Box**	5	1.05	0.21	0.59	0.70
**Cultivar**	7	22.38	3.20	9.09	< 0.001
**Residuals**	35	12.31	0.35		

As the interaction term between ‘cultivar’ and ‘date’ was highly significant, the effects of ‘box’ and ‘cultivar’ on the ‘number of eggs per gram of fruit’ was also analysed with 2-way ANOVAs for each single date. The ‘number of eggs per gram of fruit’ was log-transformed (log[x+1]).

### 3.3 Metabolomic analysis of the skin of the most and least attractive grape cultivars

**Figure 3:**
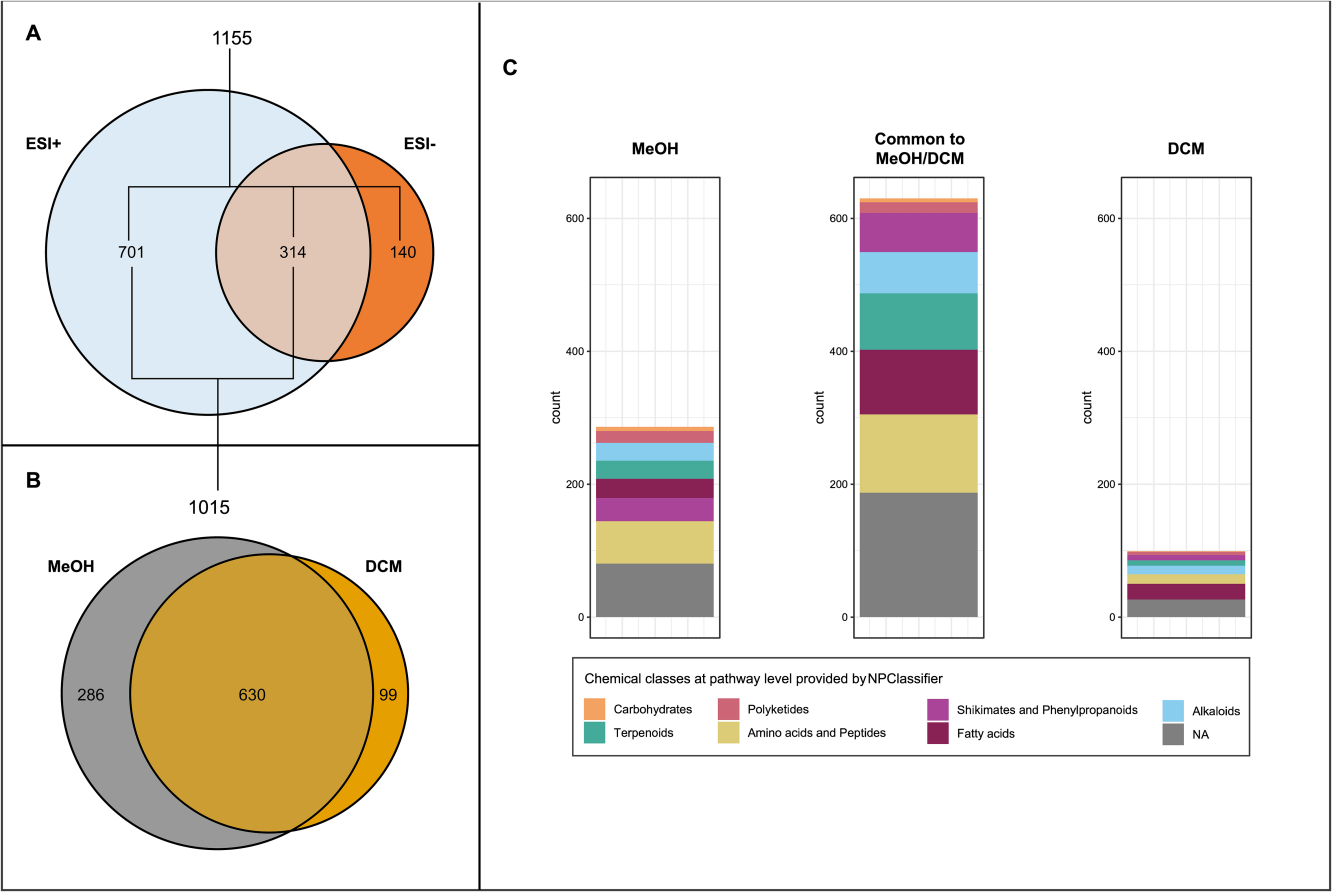
**(A)** Venn diagram of features detected in positive (ESI+) and negative (ESI-) ionisation modes in the four grape skin cultivars extracts at the four sampling dates. **(B)** Venn diagram of features detected in methanolic (MeOH) and dichloromethane (DCM) extracts in positive ionisation mode (ESI+). **(C)** Features’ chemical class distribution at pathway level provided by NPClassifier detected in MeOH and DCM extracts.

Based on their close genetic relatedness (**Table 1**) and the attractiveness results obtained in the field survey as well as the multiple-choice laboratory experiment, the two highly attractive red grape cultivars Gamay précoce and Mara as well as the two unattractive red cultivars Gamaret and Galotta were selected to search for metabolomic markers in their berry skins. Considering the broad variety of metabolites that could be involved in the interaction between grape berries and insects, a two-step extraction protocol using dichloromethane (DCM) and methanol (MeOH) was employed using UHPLC-PDA-CAD-HRMS in both positive (ESI+) and negative (ESI-) electrospray ionization modes. 1’155 features were retrieved (given *m/z* at given retention time), of which 85-90% were found with the ESI+ mode in MeOH extracts (**Figure 3A**). Features annotated through SIRIUS pipeline were categorized into five chemical classes according to NPClassifier (Kim et al., 2021) namely peptides, fatty acids, phenylpropanoids, terpenoids, and alkaloids. The retrieved features showed a broad chemical coverage without any class being over-represented (**Figure 3B**).

### 3.4 Main effects contribution to the total variability

AMOPLS models of ESI+ datasets for both extracts (MeOH and DCM) were the easiest to interpret and will be detailed hereafter. For the ESI+/MeOH dataset the grape ‘cultivar’ accounted for 10.2% of the total variability, whereas sampling ‘date’ generated 17.5% of the variance, both were highly significant (**Table 3A**). Although the interaction between the two factors explained 18.0% of the variation, it was of no statistical significance indicating that metabolic changes in the berry skins of the four cultivars over the four sampling dates were not consistent. This was further highlighted by the residuals, which accounted for 54.3% of the total variability suggesting that additional parameters affected the metabolomics dataset. Similar results were observed for the ESI+/DCM dataset, with highly significant effects for ‘cultivar’ and ‘date’ accounting for 9.5% and 15.3% of the total variability, respectively (**Table 3B**). As for the ESI+/MeOH extract, the interaction term ‘cultivar×date’ had no significant effect on metabolomics profiles.

**Table 3:** Relative variability and block contributions of the AMOPLS analysis of ESI+ metabolomic dataset of (A) MeOH and (B) DCM grape skin extracts from the four cultivars Gamay précoce, Mara, Gamaret and Galotta across the four sampling dates.

A MeOH extracts									
Effect	RSS	*p*-value	Block contributions						
			tp1	tp2	tp3	tp4	tp5	tp6	to
**Cultivar**	10.2%	<0.001	4%	**82%**	6%	12%	13%	**54%**	25%
**Date**	17.5%	<0.001	**87%**	5%	**81%**	10%	11%	13%	22%
**Cultivar×Date**	18.0%	0.792	4%	6%	6%	**65%**	**60%**	15%	24%
**Residuals**	54.3%	N/A	5%	7%	7%	14%	15%	18%	29%

B DCM extracts									
Effect	RSS	*p*-value	Block contributions						
			tp1	tp2	tp3	tp4	tp5	tp6	to
**Cultivar**	9.5%	<0.001	4%	6%	9%	**61%**	11%	**65%**	25%
**Date**	15.3%	<0.001	**89%**	5%	**74%**	12%	10%	11%	22%
**Cultivar×Date**	19.5%	0.373	3%	**81%**	8%	12%	**66%**	11%	24%
**Residuals**	55.7%	N/A	4%	7%	10%	15%	13%	14%	29%

RSS, Relative sum of squares; tp1-6, predictive components; to, orthogonal component. Values in bold contributed the strongest to the corresponding predictive component.

**Figure 4:**
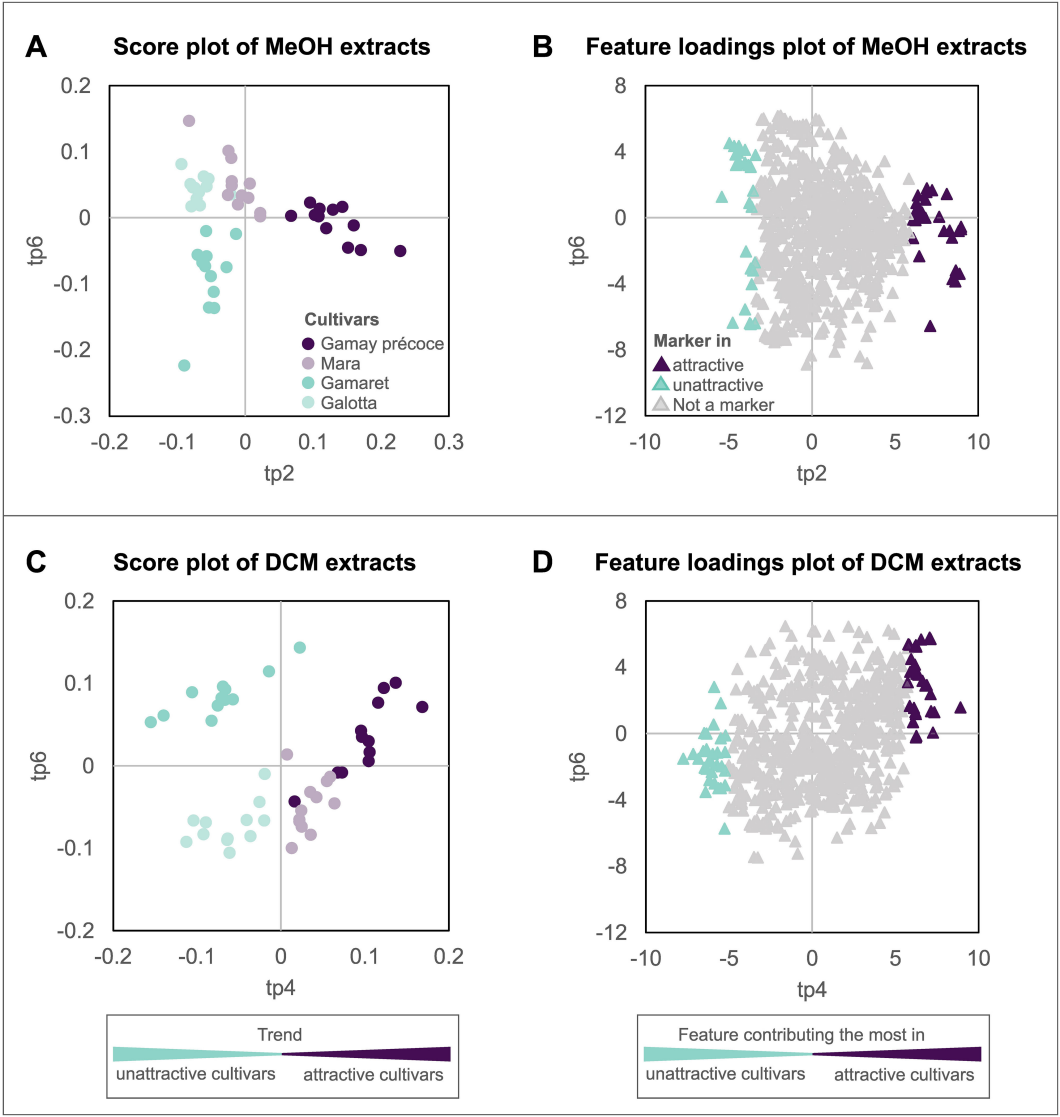
AMOPLS of sample score plots and feature loading plots of the first predictive components (tp) exhibiting the strongest contribution to the factor ‘cultivar’. Considering the ESI+/MeOH dataset, grape cultivars were best separated **(A)** by the predictive component tp2, while for the ESI+/DCM dataset, grape cultivars were separated **(C)** using tp4. The feature loading plots **(B)** and **(D)** display the contribution of each feature to the predictive components of the model.

For a better understanding how ‘cultivar’ affects the oviposition choice of *D. suzukii*, the two AMOPLS models were used to highlight metabolomic markers (annotated features) related to the attractiveness of the four grape cultivars. Our attention, therefore, concentrated on the first predictive components strongly related to ‘cultivar’ in the two AMOPLS models. Whereas ‘cultivar’ contributed 82% to tp2 in the ESI+/MeOH dataset (**Table 3A**), tp4 was considerably affected by ‘cultivar’ for the ESI+/DCM dataset (**Table 3B**). These two predictive components were consequently inspected for the distribution of attractive and unattractive cultivars along the axes (**Figures 4A, C**). For tp2 of the ESI+/MeOH dataset a strong discrimination was observed between the ‘attractive’ Gamay précoce and the ‘unattractive’ cultivars Gamaret and Galotta, while the ‘attractive’ Mara grouped in-between (**Figure 4A**). The two unattractive cultivars were associated with negative values in the loadings plot, whereas the attractive cultivars had positive loading values (**Figure 4B**). In the ESI+/DCM dataset, sample grouping was even clearer with a better clustering of ‘unattractive’ and ‘attractive’ cultivars along the predictive component tp4 (**Figure 4C**). Once again, a left-right splitting of samples was observed on the score and loading plot with negative values for the ‘unattractive’ and positive ones for the ‘attractive’ cultivars (**Figure 4D**). Unlike the PCA (**Supplementary Figure S2**), these supervised AMOPLS analyses allowed sample discrimination according to their attractiveness.

The two loading plots (**Figures 4B, D**) were then used to highlight individual features correlated to grape attractiveness toward *D. suzukii*. The thirty features with the most extreme positive and negative values along tp2 and tp4 axes were consequently selected with a total of 120 features. They were then carefully annotated by determining, as far as possible, their molecular formula, chemical class and putative structure combining the state-of-the-art computational tools GNPS, SIRIUS and timaR (Wang et al., 2016; Dührkop et al., 2019; Rutz et al., 2019).

### 3.5 Molecular network of grape skin extracts and attractiveness markers

**Figure 5:**
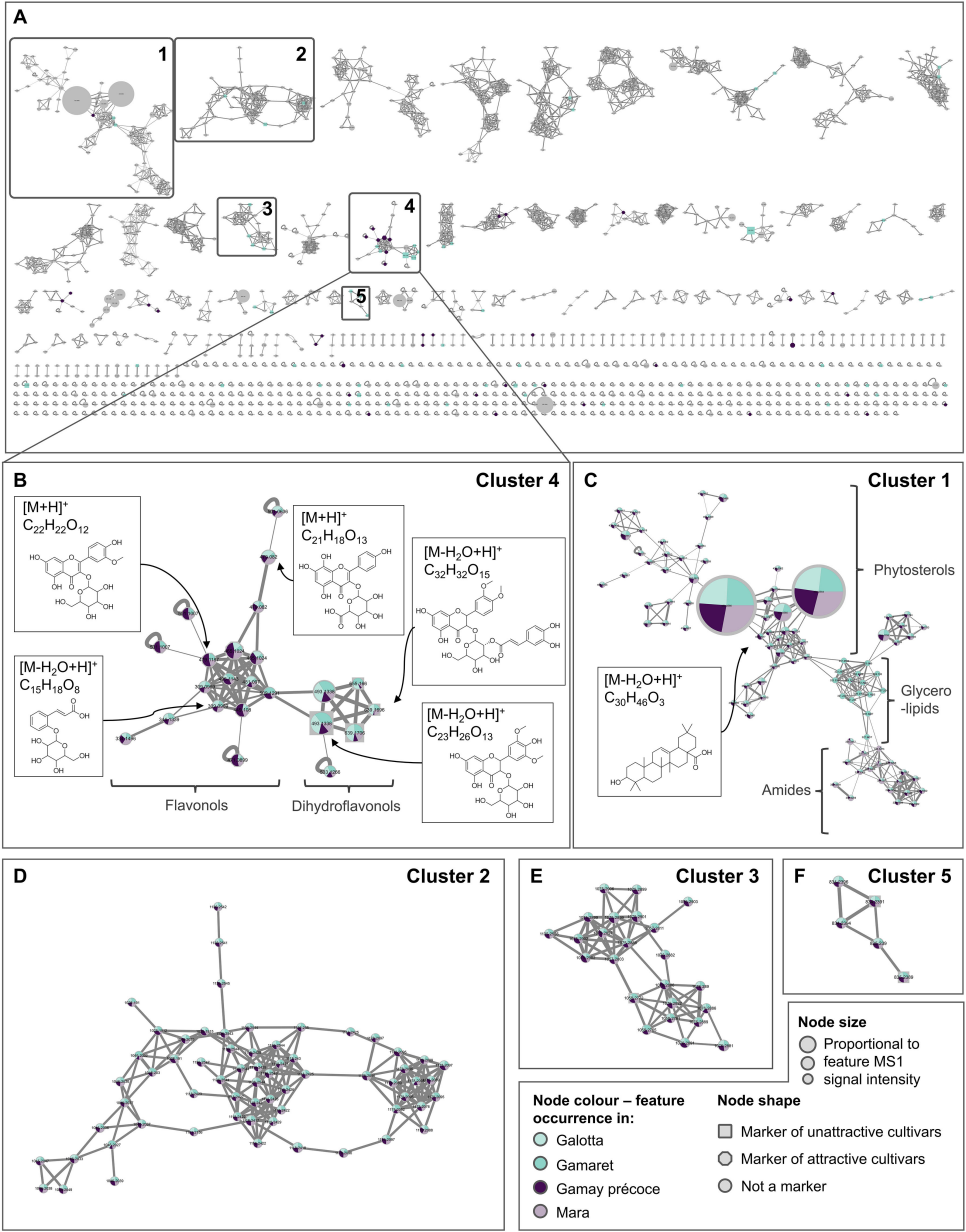
**(A)** Ion identity molecular network (IIMN) of features detected in methanolic and dichloromethane grape skin extracts from the four grape cultivars in positive ionisation mode (ESI+). Clusters 1 to 5 are emphasized by a frame as they stand out by including at least three features highlighted by the AMOPLS analyses as potential markers related to *D suzukii* attractiveness. **(B)** Zoom on cluster 4 connecting features annotated as flavonoids and phenylpropanoids derivatives. Features connected on the left side of the cluster were tentatively annotated as flavonols, while the one on the right side as dihydroflavonols. Compound structures are given for illustrative purposes. **(C)** Cluster 1 includes features tentatively annotated to different lipid classes, including phytosterols, glycerolipids and amides. **(D–F)** Zoom over clusters 2, 3 and 5 including high molecular weight features present in low abundance across samples and for which no reliable annotation was obtained. Node sizes account for combined MS intensities of each feature at the four sampling dates for the four grape cultivars. Pie charts within each node indicate the distribution of the combined MS intensities of each feature on the four sampling dates for the four grape cultivars. Node shapes indicate whether the feature was a marker of attractive cultivars (octagon), unattractive cultivars (square) or not identified as a marker (circle) according to the AMOPLS analyses. Enlarged figures for cluster 1 and 4 are available in **Supplementary Figures S4A, B**, while chemical classes of single nodes identified as markers are displayed in **Supplementary Figure S7**.

The discriminative features named as candidates were additionally analysed using a GNPS feature based ion identity molecular network (IIMN) that was built on all detected features in order to gain a better understanding of the spectral relationships and chemical relatedness. This IIMN was built by processing the MS/MS spectra of the 1’015 features identified in the ESI+ dataset and calculating the MS/MS structural relatedness among features thereby allowing features with related chemical scaffolds to cluster together (**Figure 5A**). Features having similar ion peak shapes, retention time and characteristic ion mass differences (e.g., adducts) were clustered together using this pipeline. More than 62% of the features aggregated into 78 different clusters of at least 3 nodes or more. The 120 features previously identified by the AMOPLS analyses resulted in 106 unique markers (**Supplementary Table S1**) and 14 features were found in common between the MeOH and DCM datasets. Among these 106 markers, 63 aggregated in 27 different clusters, while the 43 remaining ones corresponded each to a single node (**Supplementary Figure S3**).

To assess if attractiveness could be related to a common chemical composition, our analysis focused on markers that clustered together because we assumed that compounds of the same chemical class should elicit similar physiological effects. Only five clusters, labelled as cluster 1 to 5, aggregated more than 3 markers, meaning that most of the variability between ‘attractive’ and ‘unattractive’ cultivars might be explained through several heterogeneous classes of chemical entities. It is noteworthy that cluster 4 stood out, as it included 13 markers over 23 nodes (**Figure 5B**). The corresponding features were annotated as glycosylated flavonoids and phenylpropanoids derivatives (**Table 4**). Independently from the molecular network, the UV PDA chromatograms were acquired, and the resulting spectra allowed the identification of numerous flavonoids from the chemical group of flavonols and dihydroflavonols on the left and right side of the cluster, respectively (**Figure 5B**; **Supplementary Figure S4**). The pie charts within each node of this cluster display feature abundances for each grape cultivar. The analysis of the abundance ratio within the nodes annotated as dihydroflavonols glycosides highlighted that such compounds were considerably more abundant in the two ‘unattractive’ than in the two ‘attractive’ cultivars. Among these dihydroflavonols, three of them were esterified on the hexose moiety with either hydroxycinnamic acid (**10**, **97**) or caffeic acid (**71**), whereas one was non-esterified (**35**) (**Table 4**). Additionally, flavonols were considerably more abundant in the two ‘attractive’ compared to the two ‘unattractive’ cultivars. Specifically, most of these identified dihydroflavonols and flavonols were methoxylated at one or two positions. Further inspection of the CAD and UV traces indicated that they were among the major metabolites in the grape skin extracts (**Supplementary Figure S5**). A more detailed analysis of the feature intensities of these dihydroflavonols and flavonols over the sampling period showed that they all tended to accumulate over time (**Supplementary Figure S6**). At each of the dates, the ratio trends observed between the ‘attractive’ and ‘unattractive’ cultivars remained similar and well in line with the mean values for the four dates represented in the pie charts of the nodes (**Figures 5B–F**). This indicates that there are consistent and real constitutive differences between the two ‘attractive’ and ‘unattractive’ cultivars in their berry skin composition independent of the sampling date.

**Table 4:** Feature annotations of cluster 4 from the ion identity molecular network.

ID	*m/z*	t_R_ (min)	Adduct	Formula	Generic structure name	Marker of
**10**	639.1696	0.73	[M-H_2_O+H]^+^	C_32_H_32_O_15_	Trihydroxy-dimethoxydihydroflavonol-hexoside-hydroxycinnamic acid	unattractive
**39**	493.1338	1.13	[M-H_2_O+H]^+^	C_23_H_26_O_13_	Trihydroxy-dimethoxydihydroflavonol-hexoside	
**35**	493.1338	1.13	[M-H_2_O+H]^+^	C_23_H_26_O_13_	Trihydroxy-dimethoxydihydroflavonol-hexoside	unattractive
**37**	533.1266	1.13	[M+Na]^+^	C_23_H_26_O_13_	Trihydroxy-dimethoxydihydroflavonol-hexoside	
**71**	655.1660	1.62	[M-H_2_O+H]^+^	C_32_H_30_O_15_	Dihydroxy-dimethoxydihydroflavonol-hexoside-caffeic acid	unattractive
**97**	639.1706	1.79	[M-H_2_O+H]^+^	C_32_H_32_O_15_	Trihydroxy-dimethoxydihydroflavonol-hexoside-hydroxycinnamic acid	unattractive
**8**	334.1496	0.71	[M+NH_4_]^+^	C_14_H_20_O_8_	Phenylethanoidal (C6-C2) glycosides	
**22**	344.1340	1.00	[M+NH_4_]^+^	C_15_H_18_O_8_	Hexosyl hydroxycinnamic acid (C6-C3)	
**26**	309.0969	1.04	[M-H_2_O+H]^+^	C_15_H_18_O_8_	Hexosyl hydroxycinnamic acid (C6-C3)	attractive
**32**	309.0969	1.05	[M-H_2_O+H]^+^	C_15_H_18_O_8_	Hexosyl hydroxycinnamic acid (C6-C3)	attractive
**44**	481.0970	1.30	[M+H]^+^	C_21_H_20_O_13_	Pentahydroxyflavonol-hexoside	
**51**	479.0819	1.44	[M+H]^+^	C_21_H_18_O_13_	Pentahydroxyflavonol-hexoside	
**55**	501.0637	1.48	[M+Na]^+^	C_21_H_18_O_13_	Tetrahydroxyflavonol-Glucuronopyranoside	
**53**	465.1026	1.48	[M+H]^+^	C_21_H_20_O_12_	Tetrahydroxyflavonol-hexoside	attractive
**56**	479.0819	1.48	[M+H]^+^	C_21_H_18_O_13_	Tetrahydroxyflavonol-Glucuronopyranoside	
**62**	465.1027	1.49	[M+H]^+^	C_21_H_20_O_12_	Tetrahydroxyflavonol-hexoside	attractive
**64**	495.1146	1.51	[M+H]^+^	C_22_H_22_O_13_	Tetrahydroxy-methoxyflavonol-hexoside	
**78**	471.0899	1.65	[M+Na]^+^	C_21_H_20_O_11_	Trihydroxyflavonol-hexoside	attractive
**80**	449.1080	1.66	[M+H]^+^	C_21_H_20_O_11_	Trihydroxyflavonol-hexoside	attractive
**74**	501.1007	1.70	[M+Na]^+^	C_22_H_22_O_12_	Trihydroxy-methoxyflavonol-hexoside	attractive
**87**	509.1291	1.70	[M+H]^+^	C_23_H_24_O_13_	Trihydroxy-dimethoxyflavonol-hexoside	
**88**	479.1186	1.70	[M+H]^+^	C_22_H_22_O_12_	Trihydroxy-methoxyflavonol-hexoside	attractive
**89**	501.1007	1.70	[M+Na]^+^	C_22_H_22_O_12_	Trihydroxy-methoxyflavonol-hexoside	attractive

Markers are indicated in the last column and specified if they were abundant in the two ‘attractive’ cultivars Gamay précoce and Mara or the ‘unattractive’ Gamaret and Galotta.

Cluster 1 stood out in the analysis by its extended size of 90 nodes and its high connectivity. The features in the cluster were annotated as lipids and belonged mainly to the three subgroups phytosterols, glycerolipids and amides. Inspection of the CAD chromatograms highlighted that some of the phytosterols were major compounds in the extracts, indicated by the massive node size in the cluster (**Figure 5C**). These major phytosterols did not explain any difference in attractiveness and remained stable between cultivars. Interestingly, three minor phytosterols of cluster 1 were highlighted by the AMOPLS analysis as markers. While marker **227** was abundant in the two ‘attractive’ cultivars, the two markers **867** and **356** were rare. Firstly, since these three phytosterols are minor components of the extract and secondly, inconsistently affected attractiveness, they were not identified as easily appliable markers.

Further analysis of cluster 1 highlighted a sub-cluster (1B) corresponding to glycerolipids (**Figure 5C**). Pie charts showed a strong prevalence of these compounds in the ‘unattractive’ cultivar Gamaret, but the AMOPLS analysis did not identify these compounds as significant markers as they were only detected on the second sampling date and are probably of negligible biological relevance.

Cluster 2, 3 and 5 were the other clusters for which markers were identified by AMOPLS (**Figures 5D–F**). A close inspection of the node pie chart of all markers did not reveal any important proportional differences among cultivars. Manual inspection of the CAD chromatograms indicated that these markers were minor compounds in the extracts. Finally, no reliable annotation was obtained through the SIRIUS pipeline for the compounds of these 3 clusters. Their high molecular weight and rather high lipophilicity suggest that they are most likely lipids. As the data did not indicate a clear trend, they were not considered further.

Beside the 63 markers aggregated in 27 different clusters, 43 features highlighted by the AMOPLS corresponded to single nodes. Annotation of these markers was conducted through the SIRIUS pipeline, the GNPS platform as well as manually. When looking at the classification given by NPClassifier, flavonoids were the most represented chemical class among these markers (**Supplementary Figure S7**). A close inspection of the semi quantitative CAD signal indicated that all these single nodes were present in very low amounts in the grape skin extracts.

## 4 Discussion

Our behavioural experiments confirmed significant differences among these genetically related grape cultivars in their attractiveness to *D. suzukii*, with females showing a clear preference for laying their eggs in attractive cultivars such as Gamay précoce or Mara compared to unattractive cultivars like Gamaret or Galotta. The combination of clustering information from molecular networking and statistical AMOPLS findings with semi-quantitative CAD detection enabled us to identify flavonoids as potential markers for differences in the attractiveness of the four grape cultivars studied towards *D. suzukii*. Overall, dihydroflavonols were accumulated in unattractive grape cultivars, while attractive grape cultivars were richer in flavonols and these polyphenols were abundant metabolites in the semi-quantitative CAD analysis. In the upcoming sections, our classical behavioural experiments will be discussed and related to the existing literature and we then explore the potential impact of the identified chemical markers on the egg-laying behaviour of *D. suzukii* females. We conclude our manuscript by discussing the benefits of our advanced statistical approach for analysing metabolomic data compared to conventional methods and providing a general perspective on the potential implications of our findings.

### 4.1 Egg-laying preferences of *D. suzukii*

Our field studies as well as multiple-choice laboratory experiments confirmed that significant differences exist among grape cultivars in their attractiveness towards *D. suzukii* for egg-laying. Infestation rates increased with the ripeness of grapes. Overall, Gamay précoce and Mara showed the highest attractiveness at the last sampling date. This is in line with data collected simultaneously in surrounding, commercial vineyards (www.agrometeo.ch) and with findings from previous studies (Ioriatti et al., 2015; Kehrli and Linder, 2018; Entling et al., 2019; Tonina et al., 2020; Weißinger et al., 2019, 2021). In 2019, field observers also noted significant differences in *D. suzukii* infestation among grape cultivars in nearby vineyards with an increase in oviposition toward grape harvest and higher trap captures of adult flies at the end of the vegetation period (www.agrometeo.ch). Moreover, previous studies have demonstrated that *D. suzukii* generally prefers to lay its eggs in red cultivars over white ones (Ioriatti et al., 2015; Kehrli and Linder, 2018; Weißinger et al., 2019), that oviposition increases with grape maturity (Ioriatti et al., 2015; Kehrli and Linder, 2018) and that skin hardness affects attractiveness (Ioriatti et al., 2015; Kehrli and Linder, 2018; Entling et al., 2019; Tonina et al., 2020).

Interesting insights into the role of the epicuticular wax layers of grapes for *D. suzukii* egg-laying were gained by Weissinger et al. (2021). In their study authors manipulated the epicuticular wax layer of five grape cultivars. Although the removal of the wax layer did not affect the skin hardness of grapes, *D. suzukii* females laid up to six times more eggs in dewaxed berries than in unmanipulated ones from the same cultivar. These authors also demonstrated that the grape berry wax composition differed among cultivars, and they hypothesised that differences in the cuticular wax composition might be one reason for varietal preferences in *D. suzukii*. To determine if additional factors within the grape skin explain attractiveness, we compared the metabolomic composition of attractive and unattractive cultivars in order to identify molecular markers or chemical classes explaining behavioural preferences in *D. suzukii*. Finding molecular markers is challenging and we therefore used a supervised AMOPLS multivariate analysis in order to overcome the limitation of classical unsupervised PCA where grape maturation as the main driver of the chemical variation in the dataset (**Supplementary Figure S2**) concealed differences among cultivars. AMOPLS highlighted subsets of candidate markers, while metabolomic networks revealed their structural relationships. In the next section we discuss the biological role of markers in the context of *D. suzukii*-grape interactions.

### 4.2 Potential markers for egg-laying preferences of *D. suzukii*

Visual, tactile and organoleptic cues play major roles in the perception and attractiveness of fruits by herbivorous insects in general (Bruce and Pickett, 2011; Stensmyr et al., 2012) and *D. suzukii* in particular (Rice et al., 2016; Takahara and Takahashi, 2017). Oviposition preferences of *D. suzukii* are therefore influenced by a fruit’s visual, physical and chemical characteristics. We will now discuss how the identified potential chemical markers for the four grape cultivars might influence the visual and organoleptic perception of *D. suzukii*. Overall, dihydroflavonols were accumulated in unattractive grape cultivars, while attractive grape cultivars had more flavonols and both flavonoid types were major constituents of the extracts.

Visual factors, such as shape and colour, are initial cues that attract *D. suzukii* to a suitable host for egg-laying (Takahara and Takahashi, 2017). The attraction of *D. suzukii* towards the colour red might originate from the contrast between light and dark, its iridescence as well as its ultraviolet reflectance, rather than red’s specific hue (Little et al., 2019). Interestingly, flavonoids might impact ultraviolet reflectance. Flavonoids have specific chromophores that exhibit two main UV absorption bands: band I absorbs around 350 nm and band II around 250 nm (Taniguchi et al., 2023). Due to the 2,3-dihydro bond, band I of the dihydroflavonols absorbs at shorter wavelengths from 300-330 nm compared to flavonols from 350-385 nm (Taniguchi et al., 2023). Highlighted in a photoaxis experiment, *D. suzukii* shows a preference for UV light over higher wavelengths of 405-430 nm with a second predilection for longer wavelengths of 455-660 nm (Fountain et al., 2020). As colour opponency is assumed to influence colour perception, the accumulation of dihydroflavonols in Gamaret and Galotta might affect the wavelengths perceived by *D. suzukii*, thereby potentially repelling them from these cultivars. On the other hand, the flavonol-rich grape cultivars Gamay précoce and Mara might reflect wavelengths that attract *D. suzukii* females and promote egg-laying. Thus, the visual perception of grape cultivars might be influenced by their quantitative and qualitative composition of flavonoids. Systematically measuring the ranges of absorbed and reflected UV wavelengths among fruits could therefore provide valuable novel insights in the role of UV rays in the oviposition choices of *D. suzukii*.

If visual aspects serve as initial cues in the egg-laying behaviour, the physical and chemical characteristics of fruit skins determine oviposition by *D. suzukii* females (Reymond, 2022). The perception of the fruit surface is mediated through specific chemoreceptors (Haverkamp et al., 2018). The closely related vinegar fly *Drosophila melanogaster* Meigen has sour taste neurons, named ionotropic receptors, inside the sensilla of their legs (Benton et al., 2009). As females prefer to oviposit on acid-containing food which improves offspring performance by reducing developmental time (Fragnière et al., 2024), these taste neurons and receptors are activated by acids and regulate their behavioural choices (Chen and Amrein, 2017). Thus, initial processing of a fruit’s chemical signals is presumably essential for egg-laying. Many molecules ranging from glucosinolates (Liu et al., 2023), terpenoids (Yang et al., 2020), alkaloids to phenylpropanoids (Muthu et al., 2013) are perceived by insects as oviposition stimulants or repellents. Interestingly, an odorant-binding protein (OBP) of *D. melanogaster* was recently used to develop a bio-sensitive material for the detection of bitter molecules, such as alkaloids and flavonoids (Chen et al., 2020). The capacity of *D. suzukii* to sense flavonoids has until now not been properly demonstrated *in-vivo*. However, Dweck et al. (2021) hypothesised that a loss of bitter responses in *D. suzukii* might have contributed to its novel oviposition preference for maturing instead of degrading fruits. This is supported by observations indicating that *D. suzukii* adults have lost 20% of the bitter-sensing sensilla on their labellum and that bitter-sensing mutants of *D. melanogaster* also exhibit a shift in egg-laying behaviour from overripe toward ripe fruits (Dweck et al., 2021).

The attractive or repulsive effect of flavonoids, and more generally of phenylpropanoids, on egg-laying by adult insects and larval feeding is documented (Mierziak et al., 2014; Palma-Tenango et al., 2017; Ramaroson et al., 2022). For example, the flavanone naringenin together with the disaccharide flavonoids hesperetin-7-*O*-rutinoside and quercetin-3-*O*-rutinoside attract the swallowtail *Papilio xuthus* L. and stimulate its egg-laying on citrus plants (Ohsugi et al., 1985). Similarly, the flavanone luteolin 7-*O*-(6’’-malonyl glucoside) stimulates oviposition by the parsnip swallowtail *Papilio polyxenes* Fabricius (Feeny et al., 1988). On the other hand, the flavonoid glycoside quercetin-3-*O*-rutinoside impairs egg-laying of the cabbage butterfly *Pieris rapae* L (Tabashnik, 1987). In our study, the majority of identified flavonoids were glycosylated with the monosaccharide hexose. They were frequently esterified to hydroxycinnamic acids in the two unattractive cultivars, while glucuronidated in the two attractive cultivars. Our study enables us to highlight these structural variations among flavonoids. In order to understand their possible chemo-ecological role, their complete structural elucidation as well as their organoleptic perception by *D. suzukii* would be required.

Another particular aspect of the current work is the identification of polymethoxylated flavonoids (PMFs) and their prevalence in our unattractive cultivars. PMFs are rarely studied, but they gained interest over the last decade as a result of their numerous biological activities (Manthey and Bendele, 2008; Ortuño et al., 2011; Lakshmi and Subramanian, 2014; Chen et al., 2017). Their lipophilicity enables PMFs to interact with membrane constituents, thereby affecting the permeability of cell membranes by an increasing flux of K^+^ and a higher electrical conductivity, which can lead to severe cell malfunction and even cell death. This toxic activity highlights PMFs as promising natural antimicrobial compounds (Wu et al., 2014). In addition, PMFs may also show anticancer characteristics by inducing Ca^2+^-dependent apoptotic mechanisms (Sergeev et al., 2006). We hypothesize that the presence of PMFs could reduce egg-laying and that their biological activities could be detrimental for larval development. This is supported by the accumulation of PMFs in the two unattractive cultivars Gamaret and Galotta and the generally low emergence rate of *D. suzukii* in grapes compared to other fruits (Lee et al., 2011a; Bellamy et al., 2013; Burrack et al., 2013; Olazcuaga et al., 2019).

Our analytical strategy targeted secondary metabolites of medium polarity, revealing interesting potential markers. However, we acknowledge that these markers do not encompass the entire chemical diversity encountered in grape skins. It would be worth investigating other chemical constituent that have been reported to play a role in the grape-fly or more broadly in plant-insect interactions. An interesting class of flavonoids that we were not able to examine are anthocyanins and their glycosylated derivatives named anthocyanidins. Due to their positive charge on the C-ring oxygen and their consequently high polarity, our chosen chromatographic conditions were unfortunately not adapted to analyse these flavonoids. Yet, anthocyanins are known to be the major constituent of the epicarp of grape berries (Teixeira et al., 2014) and the principal red-pigment determining the colour of red cultivars (Revilla et al., 2001; Shahab et al., 2020). Interestingly, anthocyanins have recently been linked to plant responses and adaptation to herbivorous insects. An accumulation of anthocyanin in a gene over-expression experiment allowed to increase cotton resistance to the cotton bollworm (*Helicoverpa armigera*) and to the spider mite *Tetranychus cinnabarinus* (Li et al., 2019). Moreover, anthocyanins were reported to accumulate in plants attacked by biotic stressors such as mould, bacteria or viruses (Li and Ahammed, 2023). However, white grape cultivars such as Reichensteiner are mostly lacking anthocyanidins and they are mostly regarded with some exceptions to be unattractive to *D. suzukii* (Ioriatti et al., 2015; Kehrli and Linder, 2018; Weißinger et al., 2019). Anthocyanins might therefore play a certain role in the lower attractiveness of white varieties and they might also be implicated in the different attractiveness of red cultivars toward *D. suzukii*. Similar, volatile organic compounds (VOCs) were shown to affect the ability of *D. suzukii* to locate fruits and directly influence its oviposition and feeding behaviour (Bolton et al., 2022, 2019; Revadi et al., 2015). These VOCs emitted by plants, as well as microbial communities associated to berries, seem therefore to play a major role in the interaction of drosophilids with grapes (Becher et al., 2012; Cha et al., 2012; Rehermann et al., 2022). Developing such strategies to capture and analyse VOC remain challenging (Weingart et al., 2012). As recently reported, the chemical composition of the epicuticular wax layer of grape berries, mainly composed of triterpenoids (phytosterols), fatty acids and alkanes (Yang et al., 2021; Zhang et al., 2021), strongly affects oviposition preferences in *D. suzukii*, where dewaxed berries triggered oviposition in previously unattractive cultivars (Weißinger et al., 2021). A complementary metabolomic analysis targeting anthocyanidins, VOCs and waxes would be ideal to estimate the influence of each of these chemical factors on oviposition preference regarding our four grape cultivars. Moreover, it will also be interesting to verify in a broader study whether the observed differences in the flavonoid composition between our attractive and unattractive cultivars can be confirmed in other grape cultivars or even other fruits species. If so, these secondary metabolites might be used as potential markers for predicting the susceptibility of fruits towards *D. suzukii* infestation in the future.

## 5 Conclusions

The combination of clustering information from molecular networking and statistical AMOPLS findings with semi-quantitative CAD detection enabled us to identify that dihydroflavonols were accumulated in unattractive grape cultivars, while attractive grape cultivars were richer in flavonols and that these secondary metabolites in the grape skin might directly affect oviposition preferences of *D. suzukii*. Secondary metabolites play a crucial role in the appearance, shape, structure and organoleptic perception of grape berries but they also evolve over time along with the chemical constitution of grapes. This makes it challenging to analyse the metabolomic content and to identify molecular markers correlated with fly preferences or regulating grape attractiveness. The uniqueness of this study lies in the combination of an advanced statistical approach and the mapping of the candidate metabolites in a molecular network. AMOPLS highlighted subsets of candidate markers, while the metabolomic networks revealed structural relationships between the metabolites. As the statistical comparisons were performed on all detected features independently of their intensity range it ensured an *a priori* equal contribution of every variable to the model and even minor metabolites could potentially be identified as markers. In order to provide a semi-quantitative aspect to the obtained findings, the CAD detection information was integrated into the dataset. Based on this combined strategy, our analyses highlighted flavonoids as characteristic compounds to distinguish between attractive and unattractive grape cultivars for *D. suzukii*. These differences were observed at numerous time points during grape ripening and appear to be inherent to the grape cultivars. We, therefore, believe that our novel, integrated approach is well-suited to sensitively detect major metabolites related to multiple evolving parameters in a given biological relationship and the approach can most likely also be extended to other multifactorial changing biological systems.

## Data Availability

The data that support the findings of this study are openly available at: https://doi.org/10.5281/zenodo.11203699.
