# Supplementary material for: An advanced metabolomic approach on grape skins untangles cultivar preferences by Drosophila suzukii for oviposition
Source: Front Plant Sci. 2024 Aug 21;15:1435943. doi: 10.3389/fpls.2024.1435943 (PMC11371706; doi:10.3389/fpls.2024.1435943)
Supplement: Supplementary file 1 [file DataSheet1.docx]

**Supplementary**

**TABLE S1** Annotations table of the 106 features identified as markers from the AMOPLS analysis performed on methanolic (MeOH) and dichloromethane (DCM) extracts of berry skins of Gamay Précoce, Mara, Gamaret and Galotta varieties at the four dates of samplings date analysed in positive (ESI+) ionisation mode by UHPLC-PDA-CAD-HRMS/MS. Features from cluster 1 (lipids) are identified with the GNPS cluster n°34, while features from cluster 4 (flavonoids) are identified with the GNPS cluster n°11. NPClassifier chemical classes are given in the pathway, superclass and class columns. s. node: is for feature identified as a single node.

| ID | *m/z* | t_R_ (min) | Formula | Adduct | Pathway | Superclass | Class | Solvent | Marker in | GNPS cluster n° |
| --- | --- | --- | --- | --- | --- | --- | --- | --- | --- | --- |
| 5 | 120.0807 | 0.6 | C_8_H_9_N | [M + H]^+^ | Alkaloids | Tyrosine alkaloids | Isoquinoline alkaloids | DCM/MeOH | attractive | s. node |
| 6 | 166.0862 | 0.6 | C_9_H_11_NO_2_ | [M + H]^+^ | Amino acids and Peptides | Small peptides | Aminoacids | DCM/MeOH | attractive | s. node |
| 63 | 487.0849 | 1.49 | C_21_H_20_O_12_ | [M + Na]^+^ | Shikimates and Phenylpropanoids | Flavonoids | Flavonols | DCM/MeOH | attractive | s. node |
| 79 | 287.0550 | 1.65 | C_15_H_10_O_6_ | [M + H]^+^ | Shikimates and Phenylpropanoids | Flavonoids | Flavonols | DCM/MeOH | attractive | s. node |
| 78 | 471.0899 | 1.65 | C_21_H_20_O_11_ | [M + Na]^+^ | Shikimates and Phenylpropanoids | Flavonoids | Flavonols | DCM/MeOH | attractive | 11 |
| 80 | 449.1080 | 1.66 | C_21_H_20_O_11_ | [M + H]^+^ | Shikimates and Phenylpropanoids | Flavonoids | Flavonols | DCM/MeOH | attractive | 11 |
| 74 | 501.1007 | 1.7 | C_22_H_22_O_12_ | [M + Na]^+^ | Shikimates and Phenylpropanoids | Flavonoids | Flavonols | DCM/MeOH | attractive | 11 |
| 86 | 317.0655 | 1.7 | C_16_H_12_O_7_ | [M + H]^+^ | Shikimates and Phenylpropanoids | Flavonoids | Flavonols | DCM/MeOH | attractive | 11 |
| 89 | 501.1007 | 1.7 | C_22_H_22_O_12_ | [M + Na]^+^ | Shikimates and Phenylpropanoids | Flavonoids | Flavonols | DCM/MeOH | attractive | 11 |
| 88 | 479.1186 | 1.7 | C_22_H_22_O_12_ | [M + H]^+^ | Shikimates and Phenylpropanoids | Flavonoids | Flavonols | DCM/MeOH | attractive | 11 |
| 28 | 420.1866 | 1.14 | NA | NA | NA | NA | NA | DCM/MeOH | attractive | 62 |
| 38 | 420.1866 | 1.14 | NA | NA | NA | NA | NA | DCM/MeOH | attractive | 62 |
| 493 | 291.2681 | 5.4 | C_20_H_34_O | [M + H]^+^ | Terpenoids | Diterpenoids | Labdane diterpenoids | DCM/MeOH | attractive | 216 |
| 621 | 291.2682 | 5.73 | C_20_H_34_O | [M + H]^+^ | Terpenoids | Diterpenoids | Labdane diterpenoids | DCM/MeOH | attractive | 216 |
| 173 | 225.1485 | 3.09 | C_13_H_20_O_3_ | [M + H]^+^ | Terpenoids | Meroterpenoids | Prenyl quinone meroterpenoids | DCM | attractive | s. node |
| 53 | 465.1026 | 1.48 | C_21_H_20_O_12_ | [M + H]^+^ | Shikimates and Phenylpropanoids | Flavonoids | Flavonols | DCM | attractive | 11 |
| 1007 | 1013.2698 | 6.84 | C_48_H_54_N_4_O_14_S_2_ | [M + K]^+^ | Amino acids and Peptides | Oligopeptides | Cyclic peptides | DCM | attractive | 32 |
| 1735 | 1326.2275 | 8.4 | C_56_H_53_N_15_O_11_S_6_ | [M + Na]^+^ | Amino acids and Peptides | Oligopeptides | RiPPs Thiopeptides | DCM | attractive | 38 |
| 1750 | 1266.2609 | 8.41 | NA | NA | NA | NA | NA | DCM | attractive | 38 |
| 1781 | 1267.2618 | 8.4 | C_51_H_54_N_12_O_19_S_4_ | [M + H]^+^ | Amino acids and Peptides | Oligopeptides | RiPPs Thiopeptides | DCM | attractive | 151 |
| 1013 | 984.2383 | 6.85 | C_42_H_53_N_3_O_16_S_4_ | [M + H]^+^ | Amino acids and Peptides | Flavonoids | Polyene macrolides | DCM | attractive | 217 |
| 31 | 147.0441 | 1.05 | C_9_H_6_O_2_ | [M + H]^+^ | Polyketides | Coumarins | Simple coumarins | DCM | unattractive | s. node |
| 798 | 1074.2899 | 6.46 | C_50_H_53_N_9_O_11_S_3_ | [M + Na]^+^ | Amino acids and Peptides | Oligopeptides | RiPPs Thiopeptides | DCM | unattractive | s. node |
| 1416 | 1238.1769 | 7.63 | NA | NA | NA | NA | NA | DCM | unattractive | s. node |
| 1431 | 833.2421 | 7.7 | NA | NA | NA | NA | NA | DCM | unattractive | s. node |
| 1472 | 1072.3098 | 7.76 | NA | NA | NA | NA | NA | DCM | unattractive | s. node |
| 1471 | 1238.1762 | 7.79 | NA | NA | NA | NA | NA | DCM | unattractive | s. node |
| 1547 | 833.2418 | 7.93 | NA | NA | NA | NA | NA | DCM | unattractive | s. node |
| 1785 | 712.2346 | 8.41 | C_30_H_33_N_9_O_12_ | [M + H]^+^ | Amino acids and Peptides | Oligopeptides | Tripeptides | DCM | unattractive | s. node |
| 397 | 684.2031 | 4.93 | C_26_H_37_N_9_O_7_S_3_ | [M + H]^+^ | Amino acids and Peptides | Oligopeptides | Polyamines | DCM | unattractive | 1 |
| 662 | 684.2034 | 6.03 | C_28_H_37_N_5_O_11_S_2_ | [M + H]^+^ | Amino acids and Peptides | beta lactams | Polyamines | DCM | unattractive | 1 |
| 770 | 982.2609 | 6.3 | C_47_H_47_N_7_O_11_S_3_ | [M + H]^+^ | Amino acids and Peptides | Oligopeptides | Cyclic peptides | DCM | unattractive | 1 |
| 792 | 684.2030 | 6.44 | C_28_H_37_N_5_O_11_S_2_ | [M + H]^+^ | Amino acids and Peptides | Oligopeptides | Depsipeptides | DCM | unattractive | 1 |
| 1484 | 1027.1931 | 7.81 | NA | NA | NA | NA | NA | DCM | unattractive | 2 |
| 1524 | 1027.1938 | 7.92 | NA | NA | NA | NA | NA | DCM | unattractive | 2 |
| 26 | 309.0969 | 1.04 | C_15_H_18_O_8_ | [M - H_2_O + H]^+^ | Shikimates and Phenylpropanoids | Phenylpropanoids (C6-C3) | Cinnamic acids and derivatives | DCM | unattractive | 11 |
| 32 | 309.0969 | 1.05 | C_15_H_18_O_8_ | [M - H_2_O + H]^+^ | Shikimates and Phenylpropanoids | Phenylpropanoids (C6-C3) | Cinnamic acids and derivatives | DCM | unattractive | 11 |
| 879 | 1076.2884 | 6.63 | C_48_H_60_ClF_3_N_5_O_8_PS_3_ | [M + Na]^+^ | Alkaloids | Tryptophan alkaloids | Carboline alkaloids | DCM | unattractive | 16 |
| 888 | 1075.2892 | 6.66 | C_37_H_58_N_10_O_19_S_4_ | [M + H]^+^ | Amino acids and Peptides | Oligopeptides | Cyclic peptides | DCM | unattractive | 16 |
| 1707 | 1058.2767 | 8.37 | NA | NA | NA | NA | NA | DCM | unattractive | 16 |
| 1778 | 1058.2773 | 8.4 | NA | NA | NA | NA | NA | DCM | unattractive | 16 |
| 1492 | 1026.1945 | 7.81 | NA | NA | NA | NA | NA | DCM | unattractive | 17 |
| 1523 | 1026.1943 | 7.89 | NA | NA | NA | NA | NA | DCM | unattractive | 17 |
| 36 | 349.0900 | 1.08 | C_15_H_18_O_8_ | [M + Na]^+^ | Shikimates and Phenylpropanoids | Flavonoids | Minor lignans | DCM | unattractive | 22 |
| 1643 | 906.2602 | 8.16 | C_41_H_51_N_3_O_14_S_3_ | [M + H]^+^ | Amino acids and Peptides | Flavonoids | Linear diarylheptanoids | DCM | unattractive | 31 |
| 1720 | 906.2606 | 8.41 | C_40_H_43_F_6_N_5_O_9_S | [M + Na]^+^ | Amino acids and Peptides | Oligopeptides | Cyclic peptides | DCM | unattractive | 31 |
| 1393 | 1178.2098 | 7.58 | NA | NA | NA | NA | NA | DCM | unattractive | 36 |
| 1506 | 1028.1919 | 7.88 | NA | NA | NA | NA | NA | DCM | unattractive | 36 |
| 1705 | 1118.2424 | 8.4 | C_50_H_57_N_5_O_13_S_5_ | [M + Na]^+^ | Amino acids and Peptides | Oligopeptides | Indole diketopiperazine alkaloids | DCM | unattractive | 36 |
| 449 | 684.2031 | 4.99 | C_24_H_45_NO_15_S_3_ | [M + H]^+^ | Amino acids and Peptides | Amino acid glycosides | Glucosinolates | DCM | unattractive | 38 |
| 1758 | 982.2641 | 8.39 | C_47_H_47_N_7_O_11_S_3_ | [M + H]^+^ | Amino acids and Peptides | Oligopeptides | Cyclic peptides | DCM | unattractive | 46 |
| 1783 | 998.2972 | 8.39 | C_48_H_59_N_3_O_12_S_4_ | [M + H]^+^ | Shikimates and Phenylpropanoids | Flavonoids | Glucosinolates | DCM | unattractive | 46 |
| 1776 | 685.2037 | 8.4 | C_29_H_40_N_4_O_9_S_3_ | [M + H]^+^ | Amino acids and Peptides | Peptide alkaloids | Depsipeptides | DCM | unattractive | 99 |
| 1699 | 996.3121 | 8.38 | C_38_H_59_N_11_O_12_S_3_ | [M + K]+ | Amino acids and Peptides | Oligopeptides | Cyclic peptides | DCM | unattractive | 140 |
| 1770 | 996.3121 | 8.4 | C_42_H_57_N_7_O_15_S_3_ | [M + H]^+^ | Amino acids and Peptides | Oligopeptides | Cyclic peptides | DCM | unattractive | 140 |
| 1438 | 834.2396 | 7.72 | NA | NA | NA | NA | NA | DCM | unattractive | 176 |
| 1538 | 835.2389 | 7.92 | NA | NA | NA | NA | NA | DCM | unattractive | 176 |
| 1548 | 834.2391 | 7.93 | NA | NA | NA | NA | NA | DCM | unattractive | 176 |
| 7 | 328.1390 | 0.62 | C_15_H_21_NO_7_ | [M + H]^+^ | Amino acids and Peptides | Small peptides | Dipeptides | MeOH | attractive | s. node |
| 49 | 487.0849 | 1.43 | C_21_H_20_O_12_ | [M + Na]^+^ | Shikimates and Phenylpropanoids | Flavonoids | Flavonols | MeOH | attractive | s. node |
| 61 | 303.0499 | 1.49 | C_15_H_10_O_7_ | [M + H]^+^ | Shikimates and Phenylpropanoids | Flavonoids | Flavonols | MeOH | attractive | s. node |
| 67 | 451.1234 | 1.55 | C_21_H_22_O_11_ | [M + H]^+^ | Shikimates and Phenylpropanoids | Flavonoids | Dihydroflavonols | MeOH | attractive | s. node |
| 82 | 303.0506 | 1.62 | C_15_H_10_O_7_ | [M + H]^+^ | Shikimates and Phenylpropanoids | Flavonoids | Flavonols | MeOH | attractive | s. node |
| 159 | 227.1252 | 2.65 | NA | NA | NA | NA | NA | MeOH | attractive | s. node |
| 310 | 694.4006 | 4.18 | C_33_H_59_NO_14_ | [M + H]^+^ | Fatty acids | Glycerolipids | Glycosylmonoacylglycerols | MeOH | attractive | s. node |
| 354 | 281.1724 | 4.71 | NA | NA | NA | NA | NA | MeOH | attractive | s. node |
| 902 | 814.5602 | 6.67 | C_44_H_80_NO_10_P | [M + H]^+^ | Fatty acids | Glycerophospholipids | Oxidized glycerophospholipids | MeOH | attractive | s. node |
| 62 | 465.1027 | 1.49 | C_21_H_20_O_12_ | [M + H]^+^ | Shikimates and Phenylpropanoids | Flavonoids | Flavonols | MeOH | attractive | 11 |
| 227 | 455.3517 | 3.65 | C_30_H_46_O_3_ | [M + H]^+^ | Terpenoids | Triterpenoids | Oleanane triterpenoids | MeOH | attractive | 34 |
| 355 | 259.1904 | 4.71 | C_14_H_26_O_4_ | [M + H]^+^ | Fatty acids | Fatty esters | Wax monoesters | MeOH | attractive | 88 |
| 343 | 520.3395 | 4.56 | C_26_H_50_NO_7_P | [M + H]^+^ | Fatty acids | Glycerophospholipids | Glycerophosphocholines | MeOH | attractive | 121 |
| 340 | 478.2928 | 4.54 | C_23_H_44_NO_7_P | [M + H]^+^ | Fatty acids | Glycerophospholipids | Glycerophosphoethanolamines | MeOH | attractive | 159 |
| 705 | 986.6031 | 6.13 | C_45_H_83_N_11_O_11_S | [M + H]^+^ | Fatty acids | Glycerolipids | Diacylglycerols | MeOH | attractive | 160 |
| 837 | 824.5525 | 6.51 | C_45_H_77_NO_12_ | [M + H]^+^ | Fatty acids | Macrolides | Glycerophosphoserines | MeOH | attractive | 160 |
| 15 | 465.1017 | 0.82 | NA | NA | NA | NA | NA | MeOH | unattractive | s. node |
| 16 | 465.1017 | 0.83 | NA | NA | NA | NA | NA | MeOH | unattractive | s. node |
| 20 | 449.1080 | 0.95 | NA | NA | NA | NA | NA | MeOH | unattractive | s. node |
| 21 | 479.1185 | 0.99 | C_22_H_22_O_12_ | [M + H]^+^ | Shikimates and Phenylpropanoids | Flavonoids | Flavonols | MeOH | unattractive | s. node |
| 30 | 463.1235 | 1.11 | C_22_H_22_O_11_ | [M + H]^+^ | Shikimates and Phenylpropanoids | Flavonoids | Anthocyanidins | MeOH | unattractive | s. node |
| 42 | 507.1132 | 1.21 | NA | NA | NA | NA | NA | MeOH | unattractive | s. node |
| 47 | 491.1185 | 1.34 | NA | NA | NA | NA | NA | MeOH | unattractive | s. node |
| 48 | 521.1289 | 1.36 | C_24_H_24_O_13_ | [M + H]^+^ | Shikimates and Phenylpropanoids | Flavonoids | Flavonols | MeOH | unattractive | s. node |
| 58 | 505.1343 | 1.49 | C_24_H_24_O_12_ | [M + H]^+^ | Shikimates and Phenylpropanoids | Flavonoids | Flavones | MeOH | unattractive | s. node |
| 60 | 535.1445 | 1.49 | C_25_H_26_O_13_ | [M + H]^+^ | Shikimates and Phenylpropanoids | Flavonoids | Flavones | MeOH | unattractive | s. node |
| 65 | 611.1387 | 1.54 | C_30_H_26_O_14_ | [M + H]^+^ | Shikimates and Phenylpropanoids | Flavonoids | Flavonols | MeOH | unattractive | s. node |
| 76 | 595.1438 | 1.65 | NA | NA | NA | NA | NA | MeOH | unattractive | s. node |
| 81 | 625.1554 | 1.66 | C_31_H_28_O_14_ | [M + H]^+^ | Shikimates and Phenylpropanoids | Flavonoids | Flavonols | MeOH | unattractive | s. node |
| 95 | 679.1640 | 1.79 | C_32_H_32_O_15_ | [M + Na]^+^ | Shikimates and Phenylpropanoids | Flavonoids | Flavones | MeOH | unattractive | s. node |
| 172 | 274.2529 | 3.07 | C_19_H_31_N | [M + H]^+^ | Alkaloids | Lysine alkaloids | Piperidine alkaloids | MeOH | unattractive | s. node |
| 306 | 318.3004 | 4.04 | C_18_H_39_NO_3_ | [M + H]^+^ | Fatty acids | Sphingolipids | Sphingoid bases | MeOH | unattractive | s. node |
| 789 | 607.4189 | 6.33 | C_33_H_60_O_8_ | [M + Na]^+^ | Fatty acids | Glycerolipids | Diacylglycerols | MeOH | unattractive | s. node |
| 789 | 607.4189 | 6.33 | C_33_H_60_O_8_ | [M + Na]^+^ | Fatty acids | Glycerolipids | Diacylglycerols | MeOH | unattractive | s. node |
| 909 | 595.4186 | 6.67 | C_32_H_60_O_8_ | [M + Na]^+^ | Fatty acids | Glycerolipids | Triacylglycerols | MeOH | unattractive | s. node |
| 1298 | 714.5520 | 7.37 | NA | NA | NA | NA | NA | MeOH | unattractive | s. node |
| 1450 | 626.1761 | 7.7 | NA | NA | NA | NA | NA | MeOH | unattractive | 1 |
| 10 | 639.1696 | 0.73 | C_32_H_32_O_15_ | [M – H_2_O + H]^+^ | Shikimates and Phenylpropanoids | Flavonoids | Dihydroflavonols | MeOH | unattractive | 11 |
| 35 | 493.1338 | 1.13 | C_23_H_26_O_13_ | [M – H_2_O + H]^+^ | Shikimates and Phenylpropanoids | Flavonoids | Dihydroflavonols | MeOH | unattractive | 11 |
| 71 | 655.1660 | 1.62 | C_32_H_30_O_15_ | [M – H_2_O + H]^+^ | Shikimates and Phenylpropanoids | Flavonoids | Dihydroflavonols | MeOH | unattractive | 11 |
| 97 | 639.1706 | 1.79 | C_32_H_32_O_15_ | [M – H_2_O + H]^+^ | Shikimates and Phenylpropanoids | Flavonoids | Dihydroflavonols | MeOH | unattractive | 11 |
| 196 | 309.1114 | 3.33 | C_19_H_16_O_4_ | [M + H]^+^ | Shikimates and Phenylpropanoids | Diarylheptanoids | Linear diarylheptanoids | MeOH | unattractive | 14 |
| 59 | 553.1549 | 1.49 | C_25_H_28_O_14_ | [M + H]^+^ | Shikimates and Phenylpropanoids | Isoflavonoids | Simple coumarins | MeOH | unattractive | 22 |
| 1357 | 431.3519 | 7.54 | NA | NA | NA | NA | NA | MeOH | unattractive | 30 |
| 356 | 425.3773 | 4.73 | C_30_H_48_O | [M + H]^+^ | Terpenoids | Triterpenoids | Friedelane triterpenoids | MeOH | unattractive | 34 |
| 867 | 441.3727 | 6.57 | C_30_H_48_O_2_ | [M + H]^+^ | Terpenoids | Triterpenoids | Oleanane triterpenoids | MeOH | unattractive | 34 |
| 867 | 441.3727 | 6.57 | C_30_H_48_O_2_ | [M + H]^+^ | Terpenoids | Triterpenoids | Oleanane triterpenoids | MeOH | unattractive | 34 |
| 1737 | 1328.2268 | 8.4 | C_45_H_63_N_9_O_24_S_6_ | [M + Na]^+^ | Amino acids and Peptides | Oligopeptides | RiPPs Thiopeptides | MeOH | unattractive | 146 |


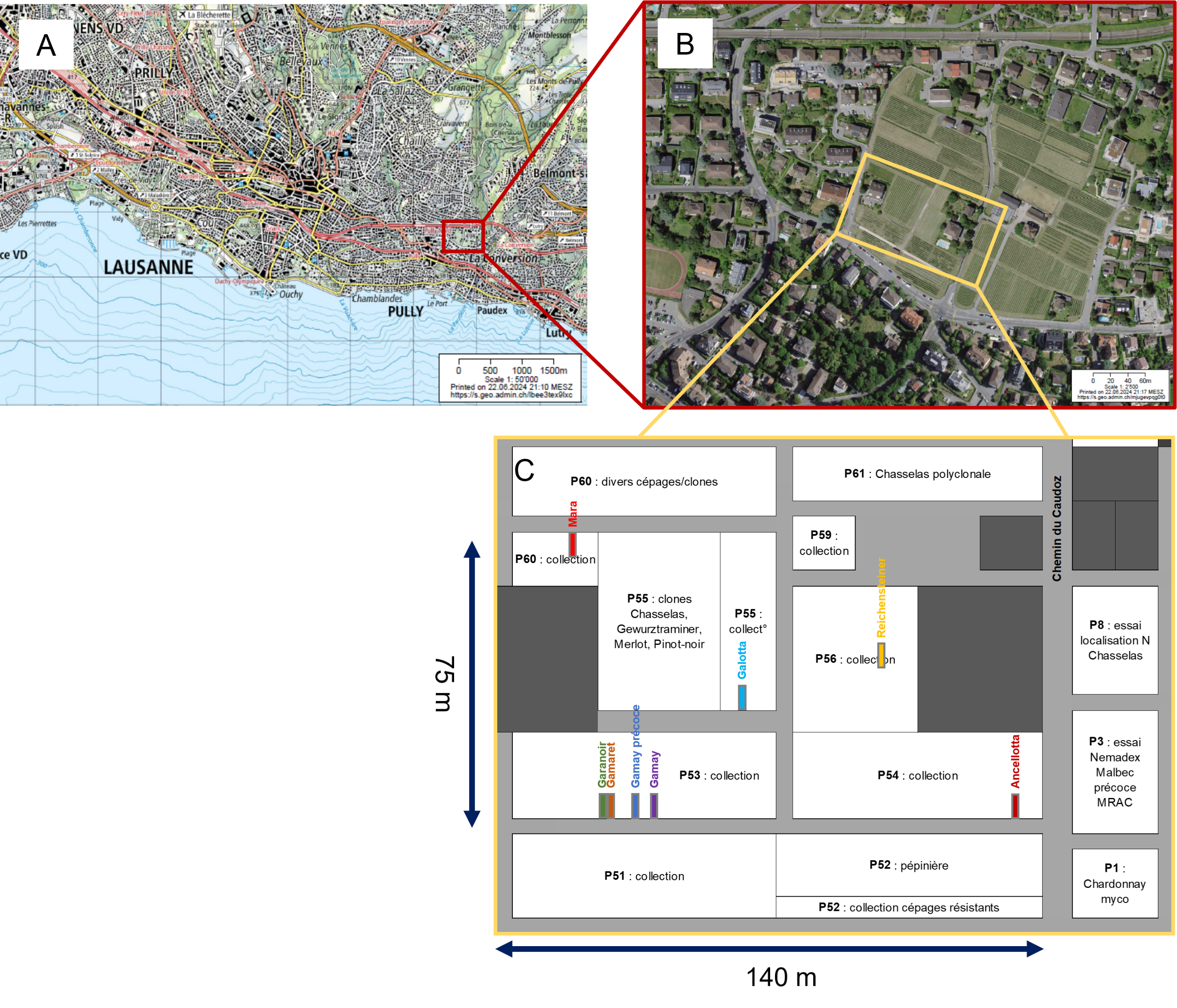


**FIGURE S1** Geographical maps of the grapevine cultivar collection of Agroscope in Pully next to Lausanne (Switzerland) (**A**) map showing the situation of the Agroscope vineyard in Pully, (**B**) aerial view on the Agroscope vineyard in Pully indicating the situation of the grapevine cultivar collection and (**C**) delineation of the situation of the studied grape cultivars within the grapevine collection. The scale in meters is indicated in the bottom of each map.


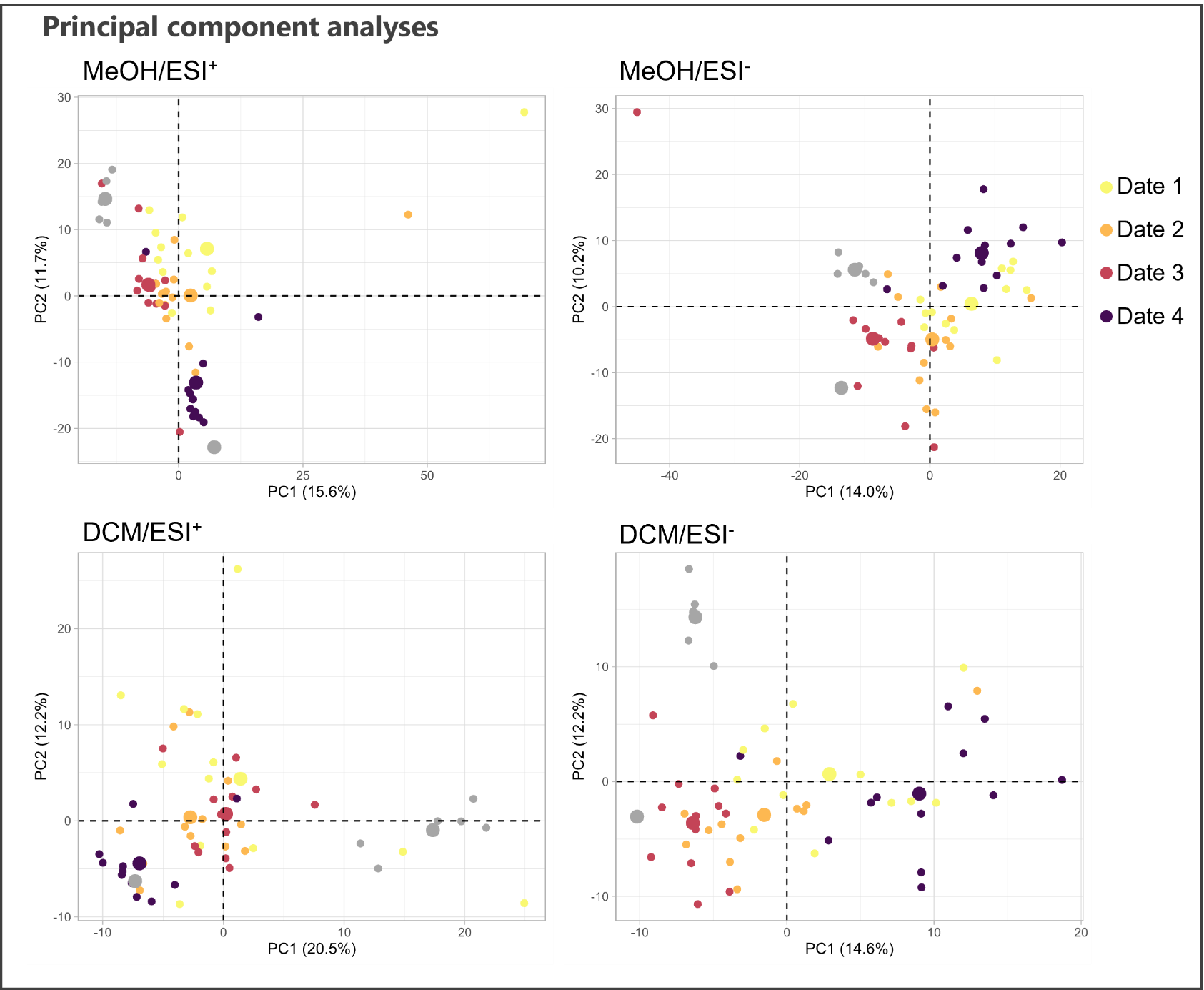


**FIGURE S2** Principal component analysis (PCA) of methanolic (MeOH) and dichloromethane (DCM) extracts of berry skins of Gamay Précoce, Mara, Gamaret and Galotta varieties at the four dates of samplings date 1 (yellow), date 2 (orange), date 3 (red) and date 4 (purple) analysed in positive (ESI+) and negative (ESI-) ionisation mode by UHPLC-PDA-CAD-HRMS/MS.


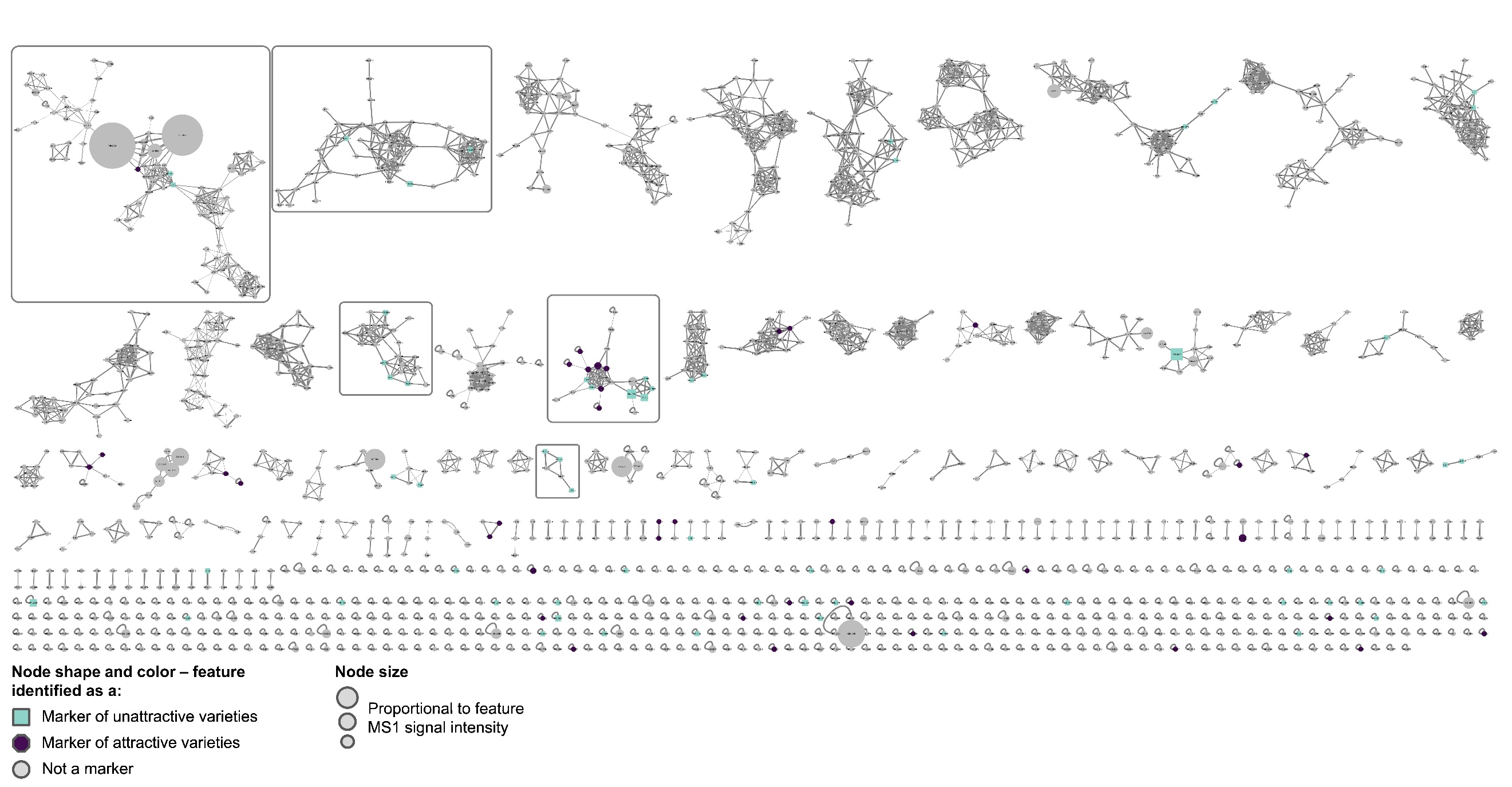


**FIGURE S3** Ion identity molecular network of the 1’155 features detected in methanolic (MeOH) and dichloromethane (DCM) extracts of berry skins of Gamay Précoce, Mara, Gamaret and Galotta varieties at the four dates of samplings date analysed in positive (ESI+) ionisation mode by UHPLC-PDA-CAD-HRMS/MS. Coloured nodes correspond to markers identified by AMOPLS analyses as overabundant in attractive varieties (purple) and unattractive varieties (green). The five clusters harbouring at least 3 markers are framed in gray.

**
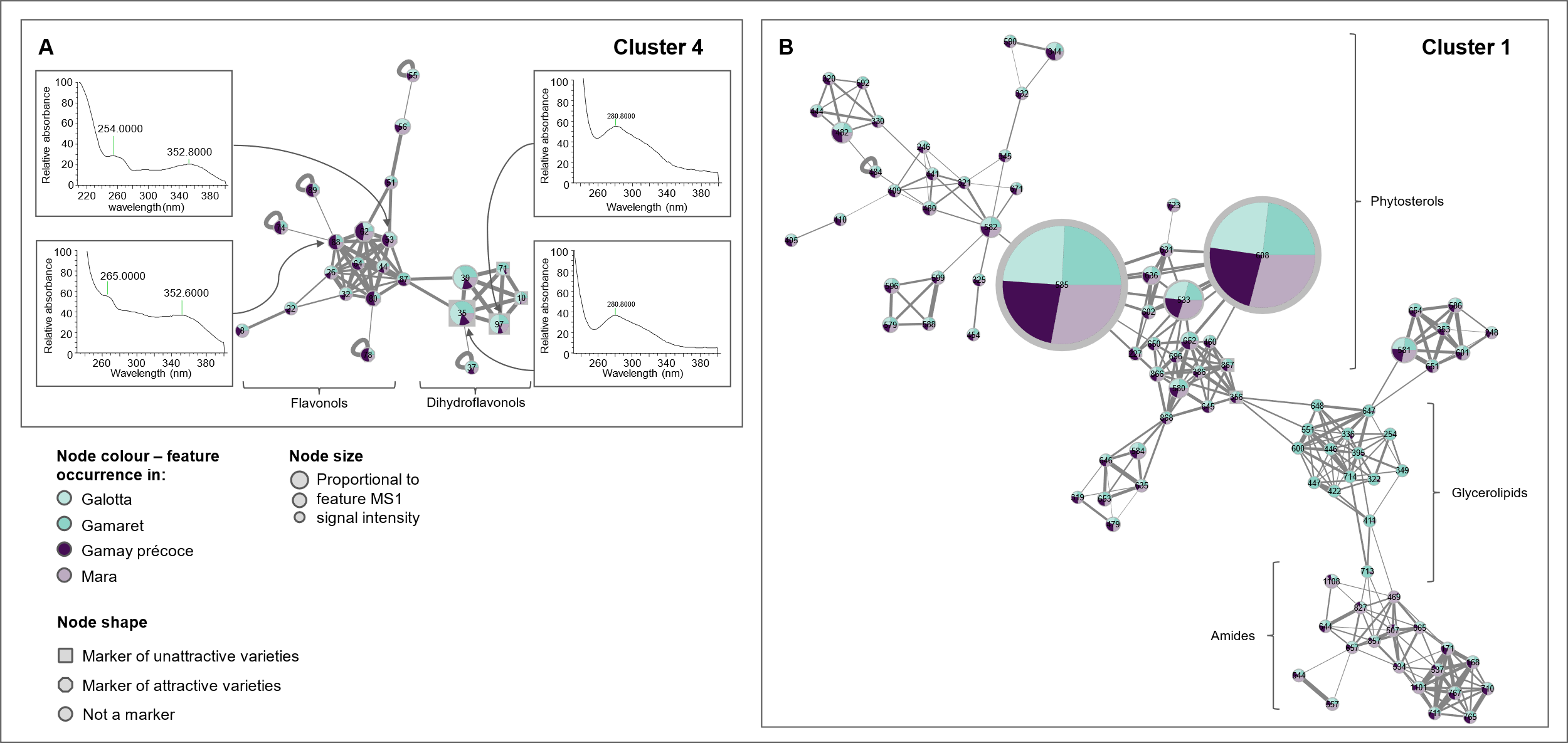
**

**FIGURE S4 (A)** Zoom over cluster 4 displaying features annotated as flavonoids and derivatives based on their exact mass, mass spectra (MS2) and UV spectra as displayed on the figure. Flavonols exhibit a specific absorption band (band I) around 350 nm while dihydroflavonols do not absorb in this wavelength spectrum. **(B)** Zoom over cluster 1 features, annotated as phytosterols, glycerolipids and amides. The two clusters belong to the ion identity molecular network (IIMN) of features detected in methanolic and dichloromethane grape skins extracts from the four grape varieties Galotta, Gamaret, Gamay précoce and Mara at the four sampling dates and analysed by UHPLC-PDA-CAD-HRMS/MS in positive ionisation mode (ESI+). Node size account for combined MS intensities of each feature at the four sampling dates for the four grape varieties. Pie charts within each node indicate the distribution of combined MS intensities of each feature at the four sampling dates for the four grape varieties. Node shape indicates whether the feature is a marker of attractive varieties (octagon), unattractive varieties (square), or not identified as a marker (circle) according to the AMOPLS analyses.


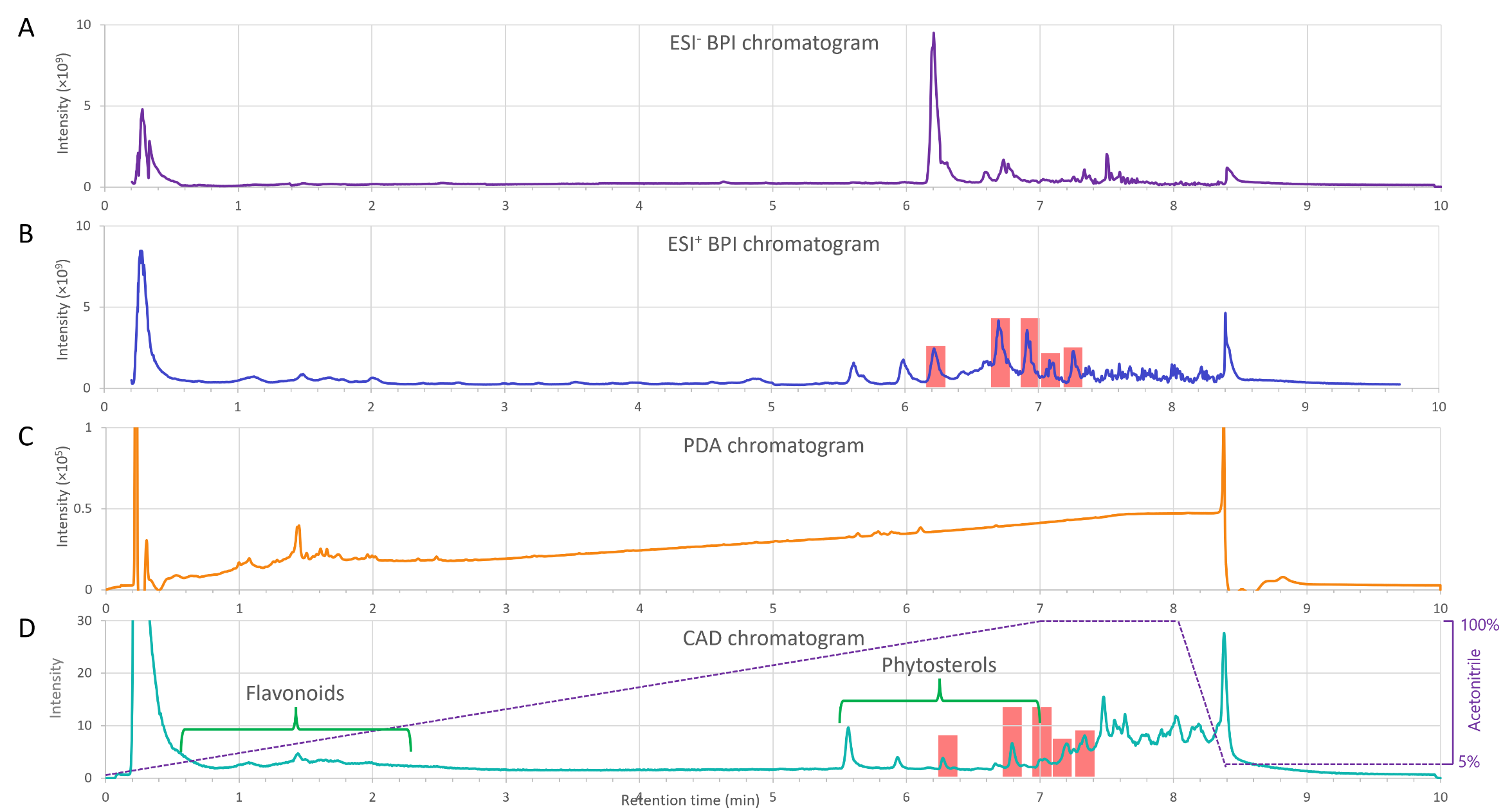


**FIGURE S5** **(A)** Base peak chromatograms in negative and **(B)** positive ionisation mode, **(C)** photodiode array detector chromatogram and **(D)** charged aerosol detector chromatogram of a mix of extracts from grape skin varieties and date of sampling used as quality control mixture. Red squares indicate contaminants.


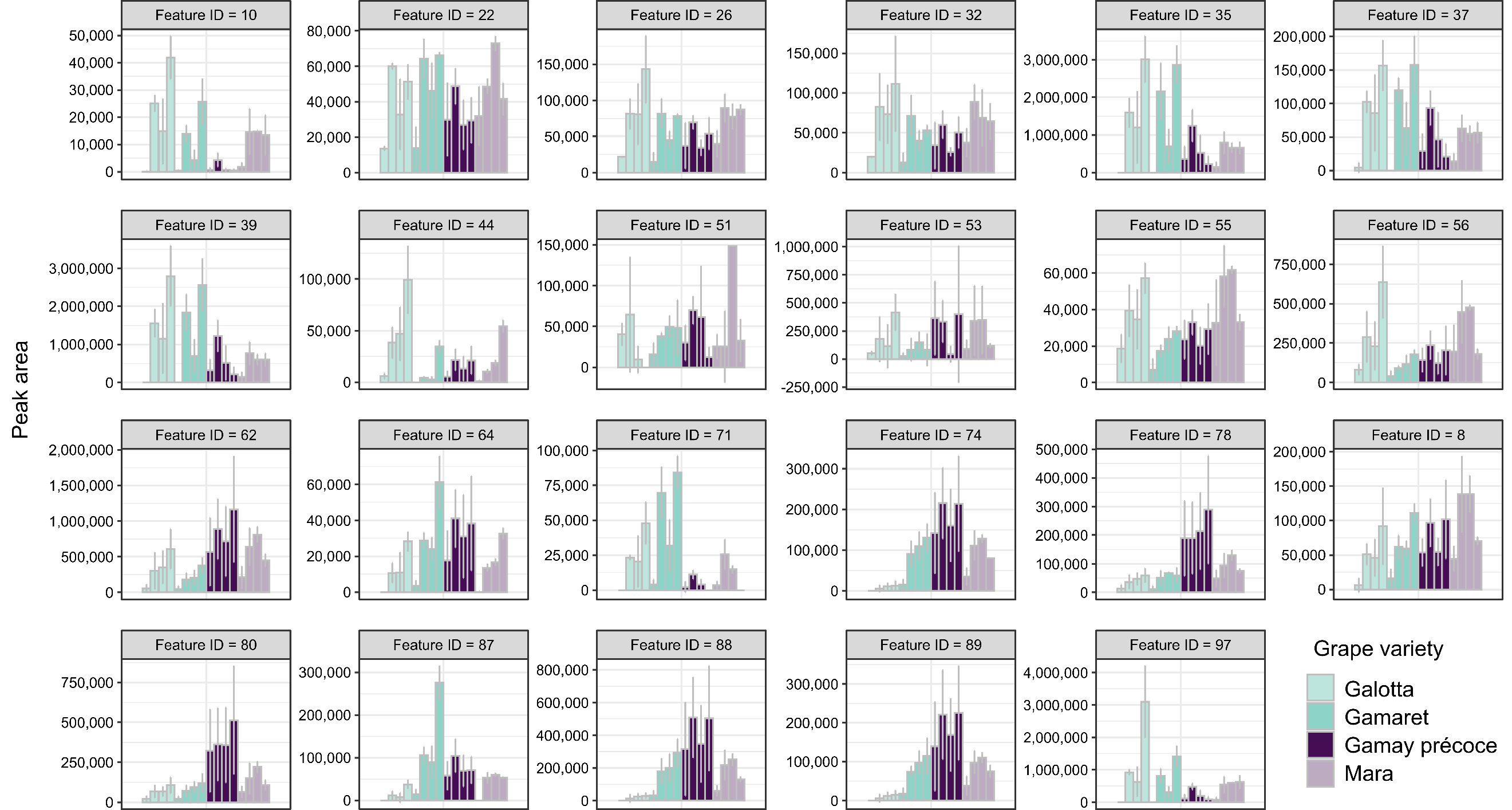


**FIGURE S6** Histograms of feature peak area from cluster 4 (flavonoids) detected in the four grape varieties Galotta, Gamaret, Gamay précoce and Mara at the four sampling dates in methanolic grape skin extracts analysed by UHPLC-PDA-CAD-HRMS/MS. For each grape variety, histograms are chronologically ordered according to the sampling date, from left to right corresponding to sampling date 1 to 4.


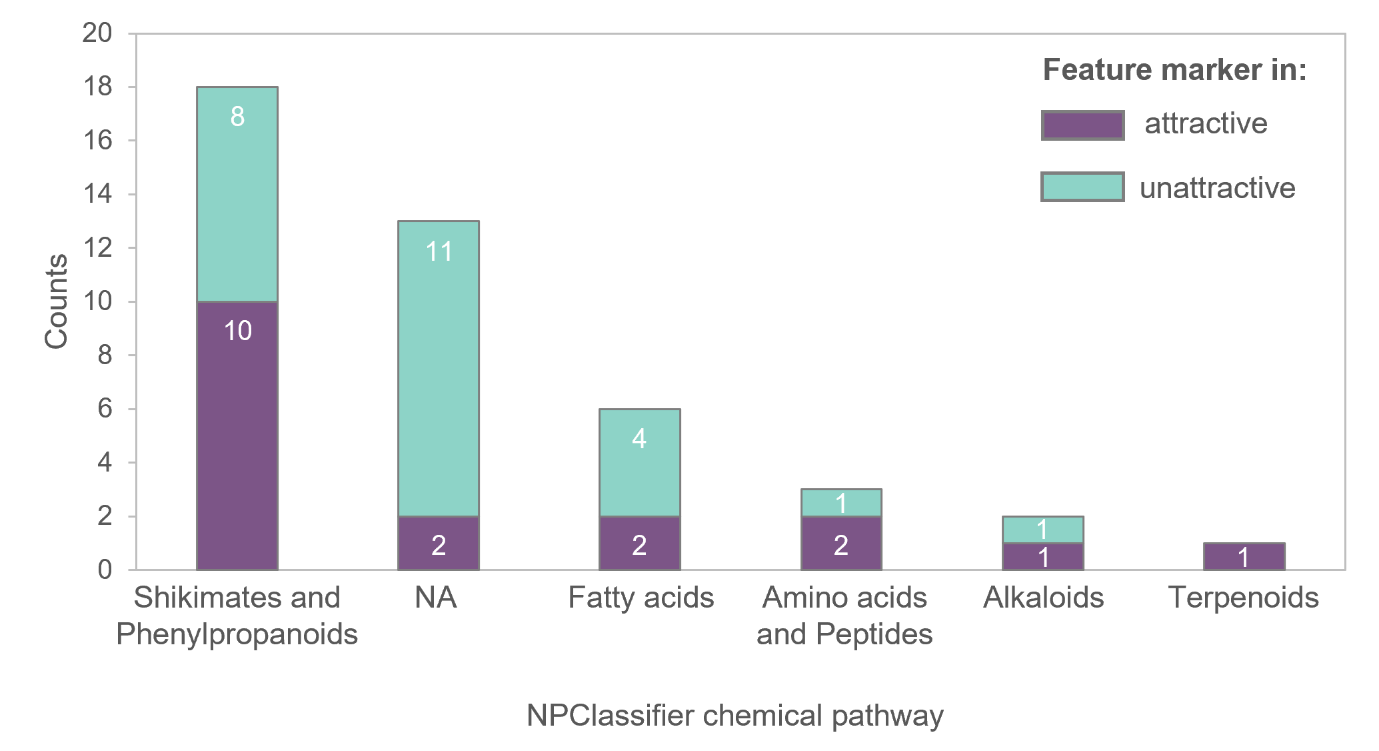


**FIGURE S7** NPClassifier chemical pathway distribution of markers identified as single nodes in the molecular network. The most represented pathway is the shikimate and phenylpropanoids pathway to which flavonoids belong.
